# Lattice Distortion‐Driven Metal Exsolution in Perovskite Oxides

**DOI:** 10.1002/advs.75870

**Published:** 2026-06-01

**Authors:** Yo Han Kim, Uchan Jeon, Hyeongwon Jeong, Bo‐Ram Won, Jeong Woo Han, Jae‐ha Myung

**Affiliations:** ^1^ Department of Materials Science and Engineering Incheon National University Incheon Republic of Korea; ^2^ Department of Materials Science and Engineering Research Institute of Advanced Materials Seoul National University Seoul Republic of Korea

**Keywords:** computational simulation, dry reforming of methane, lattice distortion, nanoparticle exsolution, solid oxide fuel cell

## Abstract

Metal‐exsolved materials have garnered significant attention in the field of heterogeneous catalysis for electrochemical and thermochemical energy conversions owing to their uniform nanoparticle dispersion and strong metal‐support socketing. Here, we propose a lattice‐engineering strategy that promotes metal exsolution by doping smaller cations into perovskite oxides, thereby inducing lattice distortion. According to computational simulations and experimental trends, lattice distortion destabilizes the perovskite lattice, lowers the energetic barrier for oxygen vacancy formation, and accelerates Ni segregation, enhancing exsolution of metallic nanoparticles. As a result, highly distorted perovskites exhibit enhanced reducibility, increased exsolved nanoparticle densities, and superior catalytic performance in electrochemical hydrogen oxidation and thermochemical dry reforming of CH_4_ and CO_2_. Moreover, the socketed structure of nanoparticles and surface basicity of oxides suppresses agglomeration and undesirable side reactions, retaining their high activity. This work highlights lattice destabilization as a driving force for promoting metal exsolution, which enables highly active and durable catalysis.

## Graphical abstract

This study elucidates the mechanism of lattice distortion‐driven exsolution through a combination of computational simulations and experimental approaches. Lattice distortion facilitates the formation of oxygen vacancies and subsequent metal segregation, thereby enhancing the exsolution behavior. This results in a higher particle density and a larger exsolution amount, which increases the density of catalytically active sites and thus promotes both electrochemical and thermochemical reaction kinetics.

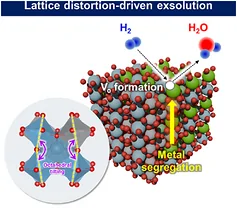

## 1 Introduction

Oxide‐supported nanoparticles play a crucial role in electrochemical and thermochemical catalysis for energy conversions such as solid oxide fuel cells (SOFCs) and reforming processes [1, 2, 3]. Their unique catalytic activity originates from exposed metal surfaces and support–metal interfaces where their density and stability determine overall performance. However, under high‐temperature and harsh carbon fuel conditions, such supported nanoparticles suffer from particle coalescence and carbon deposition, leading to the loss of active sites and eventual nanoparticle detachment from the oxide supports [4]. These intrinsic stability limitations fundamentally hinder both catalytic efficiency and long‐term durability.

The exsolution method has emerged as an alternative technique for synthesizing oxide‐supported nanocatalysts owing to the excellent uniformity, stability, and activity originating from their self‐generated emergence and distinctive architecture [5, 6, 7, 8]. In this approach, catalytically active metals are incorporated as cations into the host oxide lattice during synthesis and are subsequently exsolved as uniformly distributed nanoparticles on the oxide surface via a one‐step reduction. The exsolved nanoparticles are strongly anchored (socketed) to the oxide support, thereby enhancing metal‐support adhesion [9]. This robust architecture provides exceptional resistance to nanoparticle agglomeration, carbon coking, and sulfur poisoning, addressing critical durability issues associated with conventional nanocatalysts [10].

Squeezing out metallic nanoparticles via exsolution from structurally tolerant perovskite oxides is a promising approach for maximizing the density of catalytically active sites [11]. To enhance metal exsolution, extensive research has explored additional driving forces in perovskite systems, including crystal defects [12], lattice strain [13], phase transitions [14, 15], and external energy inputs such as plasma [16, 17], microwaves [18], and electrochemical potentials [5, 19]. These approaches rely on shifting the equilibrium, thereby increasing system instability and promoting exsolution behavior. For example, A‐site deficiency (α), a crystal defect in perovskite oxides (A_1‐α_BO_3_), induces B‐site cation enrichment and deviations from ideal stoichiometry, enhancing the exsolution of B‐site metal nanoparticles accompanied by stoichiometric stabilization [17, 20, 21]. Likewise, a strained lattice in thin film systems destabilizes the cation‐oxygen bonds, promoting the exsolution process to stabilize the lattice during reduction [13, 22]. On the other hand, lattice distortion in perovskites, which is a controllable factor of structural instability, has received comparatively little attention, and its direct role in exsolution dynamics remains poorly understood [23, 24].

**FIGURE 1:**
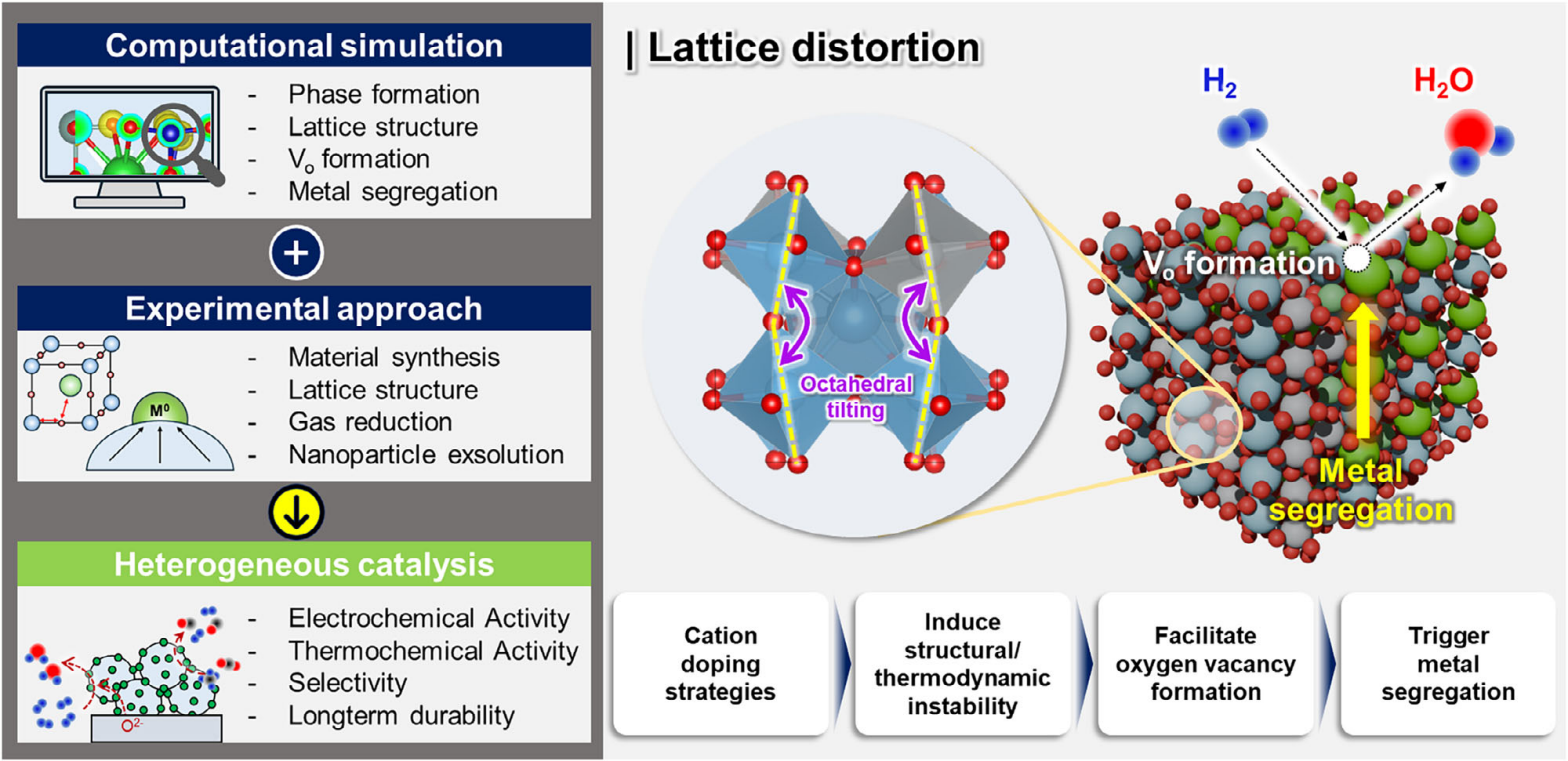
Lattice distortion‐driven exsolution strategy for enhancing catalytic activity in perovskite oxides. (Left) A combined computational simulation and experimental approach correlates phase formation, lattice structure, oxygen vacancy formation, and metal segregation with controlled A‐site cation doping. (Right) Conceptual schematic illustrating how lattice distortion in perovskite oxides is intentionally engineered to govern metal nanoparticle exsolution and catalytic functionality.

In this study, A‐site cation dopants (M = Ca^2+^, Sr^2+^, and Ba^2+^) are strategically substituted into perovskite oxides to control lattice distortion, thereby regulating metal exsolution. As illustrated in Figure 1, lattice distortion is employed as an intrinsic driving force to promote metal exsolution and enhance heterogeneous catalysis.  The effect of lattice distortion on reduction and metal exsolution is explored via a combined computational and experimental framework. Here, lattice distortion refers to the enhanced octahedral tilting and deviation from the ideal perovskite geometry. Unlike externally imposed strain engineering in thin‐film systems, this distortion originates from an intrinsic, composition‐driven thermodynamic instability of the perovskite lattice, structurally quantified by the tolerance factor (τ). We demonstrate that this cation doping strategy enables controlled lattice distortion, which facilitates oxygen vacancy formation and metal segregation during reduction, ultimately increasing the density of exsolved nanoparticles and improving catalytic performance in electrochemical hydrogen oxidation and CH_4_/CO_2_ dissociation reactions.

## 2 Results and Discussion

### 2.1 Material Design

A computational simulation was conducted based on density functional theory (DFT) to elucidate the impact of A‐site doping on lattice distortion in perovskite oxides. Before cation doping, a representative lanthanum‐based perovskite oxide, LaTiO_3_, was selected as the starting host material. The Ti cation remains stable and resistant to exsolution under reducing conditions, providing a model system that suppresses co‐exsolution and enables the clear isolation of Ni exsolution behavior [9]. To specifically control lattice distortion in titanate perovskite oxides, isovalent cations with different ionic radii (Ca^2+^: 1.34 Å, Sr^2+^: 1.44 Å, Ba^2+^: 1.61 Å) were substituted at the A‐site, while Ni was doped at the B‐site to enable metal exsolution. For computational simulations, three perovskite models were designed: La_0.75_Ca_0.25_Ni_0.25_Ti_0.75_O_3_ (LCNT), La_0.75_Sr_0.25_Ni_0.25_Ti_0.75_O_3_ (LSNT), and La_0.75_Ba_0.25_Ni_0.25_Ti_0.75_O_3_ (LBNT). DFT‐based computational simulations were conducted to elucidate how A‐site cation substitution influences lattice structure. Through each doping simulation, the thermodynamically stable configuration was selected among the possible doping configurations within a 2 × 2 × 2 supercell. After full structural relaxation, all doped structures exhibited lattice tilting induced by A‐site dopants (Figure S1). The optimized lattice parameters of LCNT, LSNT, and LBNT were 3.953, 3.969, and 3.990 Å, respectively, showing a correlation with the increasing ionic radii of the dopants (Figure S2). This trend confirms successful incorporation of dopants into the perovskite lattice and corresponding lattice expansion.

**FIGURE 2:**
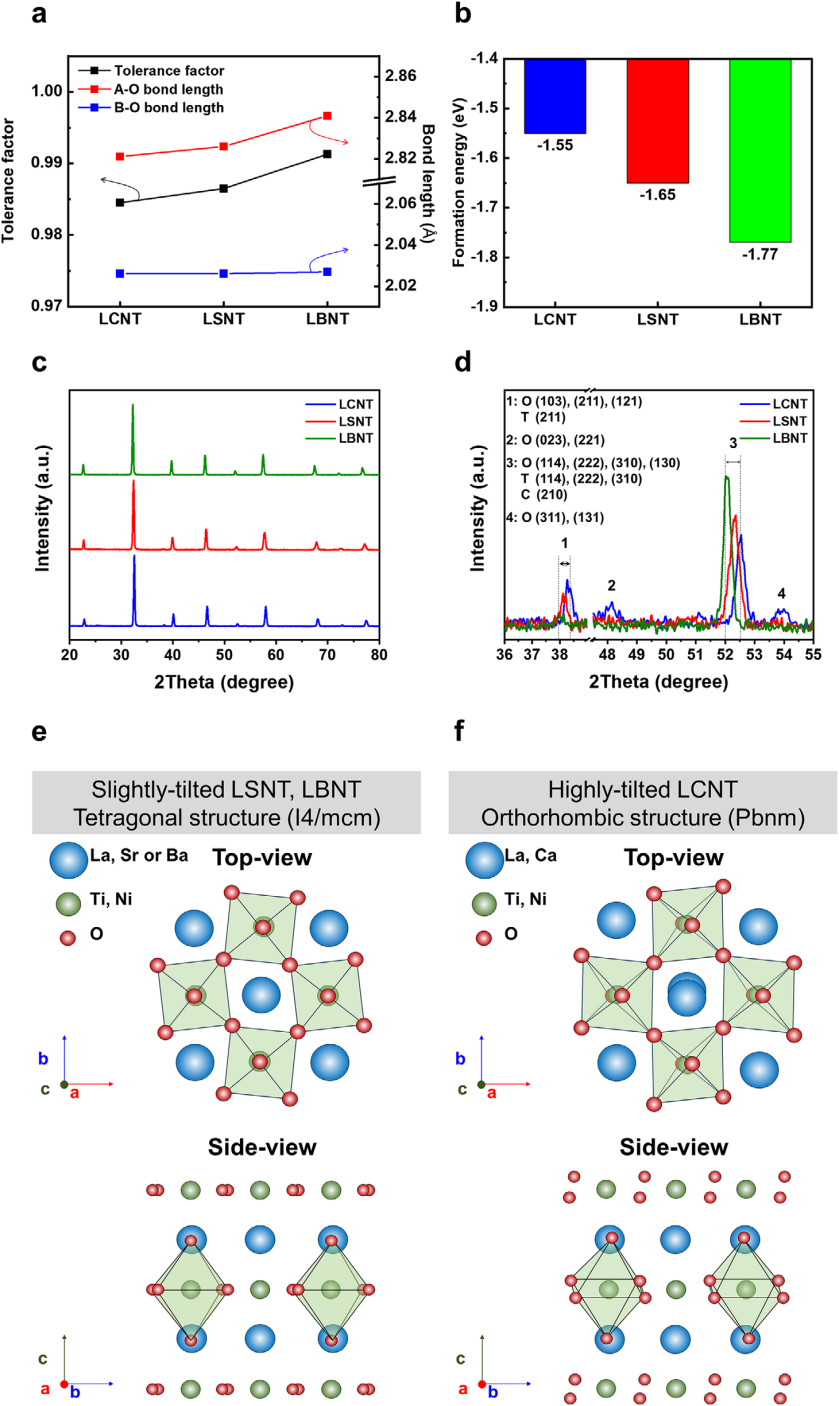
Design of lattice‐distorted perovskite oxides. (a) Structural parameters and (b) formation energies of the computational perovskite oxides doped with Ca, Sr, and Ba cations. (c) Room‐temperature powder XRD patterns of synthesized LCNT, LSNT, and LBNT. (d) Magnified XRD peaks of perovskite oxides corresponding to cubic (C, *Pm‐3m*), tetragonal (T, *I4/mcm*), and orthorhombic (O, *Pbnm*) structures. Schematic illustrations of the atomic structures of (e) tetragonal and (f) orthorhombic perovskite oxides, obtained from Rietveld‐refined crystallographic and visualized using VESTA.

The τ of perovskite oxides was employed as a structural descriptor linking octahedral tilting, lattice distortion, and structural stability. Deviations from an ideal cubic perovskite (τ = 1.0) indicate lattice distortion and reduced geometric stability, where a decrease in the A─O bond length shifts τ toward lower values [25]. LCNT exhibited the shortest A─O bond length of 2.821 Å compared to LSNT (2.826 Å) and LBNT (2.841 Å). Consistent with this trend, the calculated τ values for LCNT, LSNT, and LBNT were 0.9845, 0.9865, and 0.9913, respectively, indicating that the Ca^2+^ incorporation induces greater lattice distortion than Sr^2+^ and Ba^2+^ (Figure 2a). These results demonstrate that smaller A‐site cations shorten the A─O bond length, thereby intensifying lattice distortion in titanate perovskites. To evaluate both the thermodynamic feasibility and the relative stability of the doped structures, the formation energies for cation doping were calculated. The calculated formation energies were −1.55 eV for LCNT, −1.65 eV for LSNT, and −1.77 eV for LBNT (Figure 2b). The negative formation energies for all compositions confirm their thermodynamic feasibility for synthesis, while less negative energy values indicate reduced structural stability [26, 27, 28]. Consistent with the tendency observed in τ, LCNT exhibits the highest formation energy, indicating lower structural stability associated with the smaller A‐site dopant. These results demonstrate that smaller A‐site dopants induce stronger lattice distortion and reduce structural stability by shortening the A─O bond length.

To cross‐check the computational results using an experimental approach, Ni‐doped titanate perovskite oxides–La_0.7_Ca_0.2_Ni_0.25_Ti_0.75_O_3_, La_0.7_Sr_0.2_Ni_0.25_Ti_0.75_O_3_, and La_0.7_Ba_0.2_Ni_0.25_Ti_0.75_O_3_–were prepared with different A‐site dopant cations based on computational simulations and are referred to as LCNT, LSNT, and LBNT for simplicity. All samples were successfully synthesized as single‐phase perovskites without any secondary phases, as confirmed by powder X‐ray diffraction (XRD) analysis (Figure 2c). In the XRD patterns, some peaks corresponding to tilted perovskite structures, including tetragonal and orthorhombic symmetries, were detected at 2θ ≈ 38, 48, and 54°, as shown in Figure 2d. LSNT and LBNT crystallize in a tetragonal perovskite structure (space group *I4/mcm*), whereas LCNT exhibits an orthorhombic structure (space group *Pbnm*) with a higher degree of lattice distortion.

For quantitative structural analysis, Rietveld refinement of XRD data was performed using the identified space groups, yielding good agreement factors (χ^2^ < 3) (Figure S3). The refined cell volumes of LCNT (236.25 Å^3^), LSNT (238.87 Å^3^), and LBNT (242.10 Å^3^) increase with the ionic radius of the A‐site dopant, consistent with computational simulations, confirming the successful incorporation of cation dopants into the perovskite lattice (Table S1). Figure 2e,f presents the refined atomic structures, revealing increased octahedral tilting with decreasing A‐site ionic radius. This structural evolution is further supported by the deviation of B─O─B bond angles from the ideal 180°. LCNT exhibits a significantly smaller averaged bond angle (162.3°) compared to LSNT (169.2°) and LBNT (172.0°), indicating a higher degree of lattice distortion. This trend is in good agreement with the τ, where LCNT exhibited the lowest τ (0.973) compared to 0.995 for LSNT and 0.997 for LBNT, confirming the consistent trend in lattice distortion. This trend originates from the incorporation of the smaller Ca^2^^+^ cations at the A‐site, highlighting A‐site cation engineering as an effective approach for tuning lattice distortion in perovskite oxides.

### 2.2 Lattice Distortion‐Driven Reduction and Exsolution Behaviors

**FIGURE 3:**
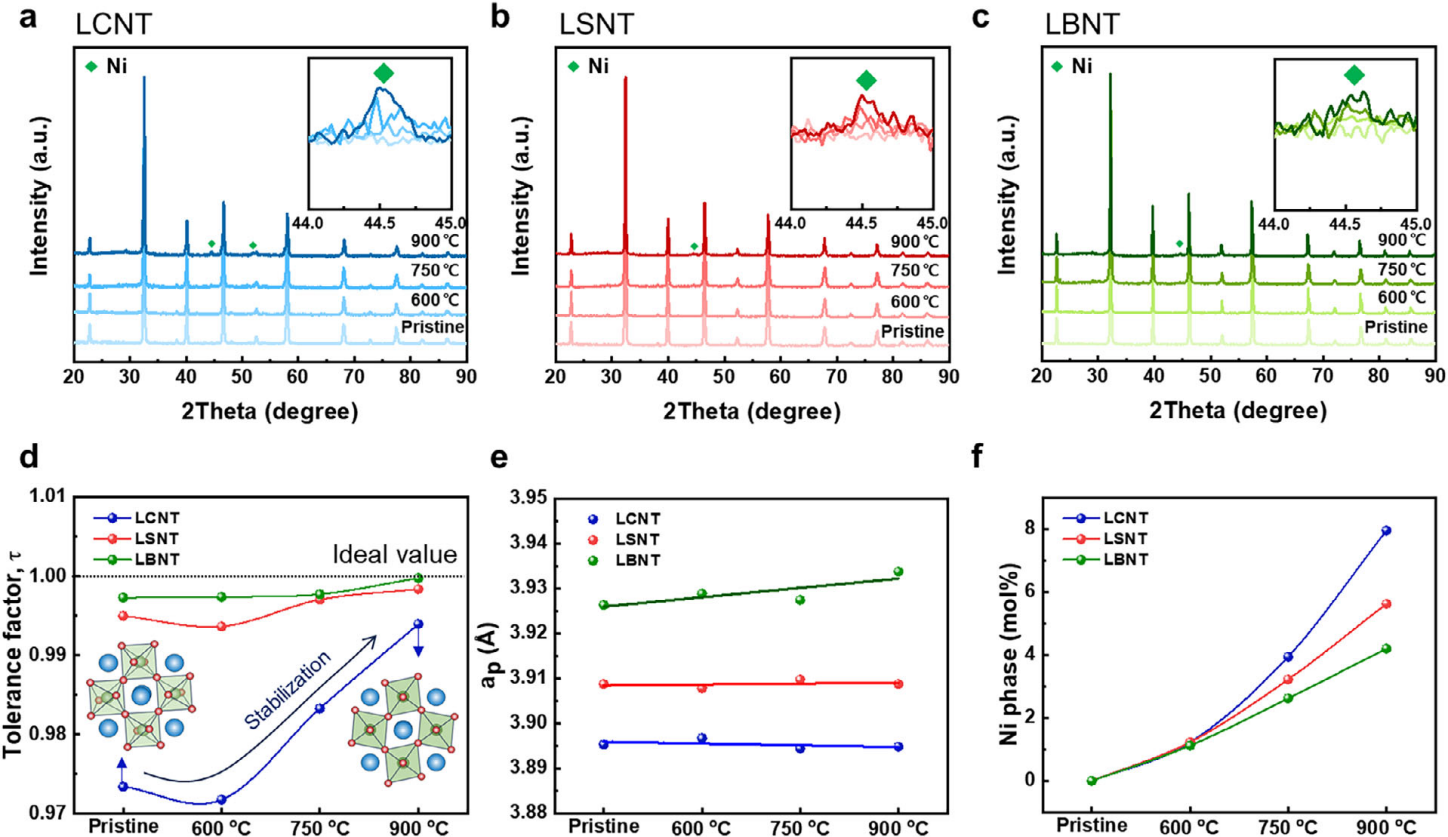
Structural evolution during gas reduction. Powder XRD patterns of (a) LCNT, (b) LSNT, and (c) LBNT before and after reduction in pure H_2_ at 600, 750 and 900°C for 20 h. (d) Tolerance factor, (e) pseudocubic lattice parameters, and (f) metallic Ni phase fractions of pristine and reduced perovskite oxides.

To track structural and phase changes during reduction, the perovskite oxides were reduced in H_2_ at 600, 750 and 900°C for 20 h, followed by XRD analysis and Rietveld refinement (Figure 3a–c, Figure S4 and Table S1). After reduction at all temperatures, a weak diffraction peak at 2θ ≈ 44.5° was observed without any additional impurity phases, confirming the exsolution of metallic Ni. During reduction, changes of τ are governed by changes in the B─O bond length (*r_B_* + *r_O_*) and A─O bond length (*r_A_* + *r_O_*), which arise from two competing processes at the B‐site. First, Ni^2+^ is reduced and exsolved as metallic Ni ($Ni_{Ti}^{2+}+2e^{-}\to Ni^{O}$) replacing the larger Ni^2+^ (0.69 Å) in the lattice with smaller Ti cations (Ti^4+^: 0.605 Å, Ti^3+^: 0.67 Å). Simultaneously, the exsolution of Ni from the B‐site creates B‐site vacancies, which can be partially compensated by the consumption of pre‐existing A‐site vacancies. This defect reorganization is associated with a shortening of the B─O bond length and a relative expansion of the A─O bond network, resulting in an increase in τ. Second, the partial reduction of Ti^4+^ to Ti^3+^ ($Ti_{Ti}^{4+}+e^{-}\to Ti_{Ti}^{3+}$) increases the B─O bond length due to the larger ionic radius of Ti^3+^, leading to a decrease in τ. Therefore, the overall change in τ during reduction depends on the relative dominance of Ni exsolution versus Ti reduction.

As the reduction temperature increased from 600 to 900°C, LCNT exhibited a significant change in τ from 0.972 to 0.995, indicating a transition from a highly distorted lattice to a more stable structure (Figure 3d). This behavior suggests that Ni exsolution in LCNT plays a dominant role in stabilizing the lattice by altering the bond lengths under high‐temperature reduction. In contrast, LSNT and LBNT showed negligible changes in τ across the same temperature range, indicating a relatively weak structural response to reduction. LCNT showed a clear lattice contraction after high‐temperature reduction, consistent with the pronounced decrease in the B─O bond length associated with Ni exsolution. In comparison, LSNT exhibited minimal changes and LBNT showed a lattice expansion despite a slight decrease in B─O bond length (Figure 3e and Table S1). The lattice expansion is dominated by changes in the A─O bond network rather than the B─O bond variations, which is particularly pronounced in the Ba‐doped oxide due to its larger A‐site ionic radius.

This enhanced Ni exsolution in LCNT at elevated temperatures is supported by the refined metallic phase fractions exsolved from the perovskite parent phase, as shown in Figure 3f. At 600°C, all samples showed similar exsolved Ni amounts (1.12–1.23 mol.%). At higher reduction temperatures (750–900°C), LCNT exhibited a significantly higher metallic Ni phase fraction (3.94–7.95 mol.%) than LSNT (3.22–5.62 mol.%) and LBNT (2.62–4.20 mol.%). Notably, no measurable secondary phases were detected after reduction (Figure S4), indicating that the perovskite lattice can accommodate the stoichiometric deviation induced by extensive Ni exsolution. This behavior is facilitated by the A‐site deficiency (∼10 mol.%), which provides vacancy sites that compensate for the stoichiometric deviation during reduction. These results suggest that the highly distorted perovskite structure promotes Ni exsolution through a lattice distortion‐driven mechanism, where metal exsolution is accompanied by structural stabilization during reduction. This behavior is analogous to strain relaxation in lattice‐strain‐driven exsolution observed in thin film model systems [13].

**FIGURE 4:**
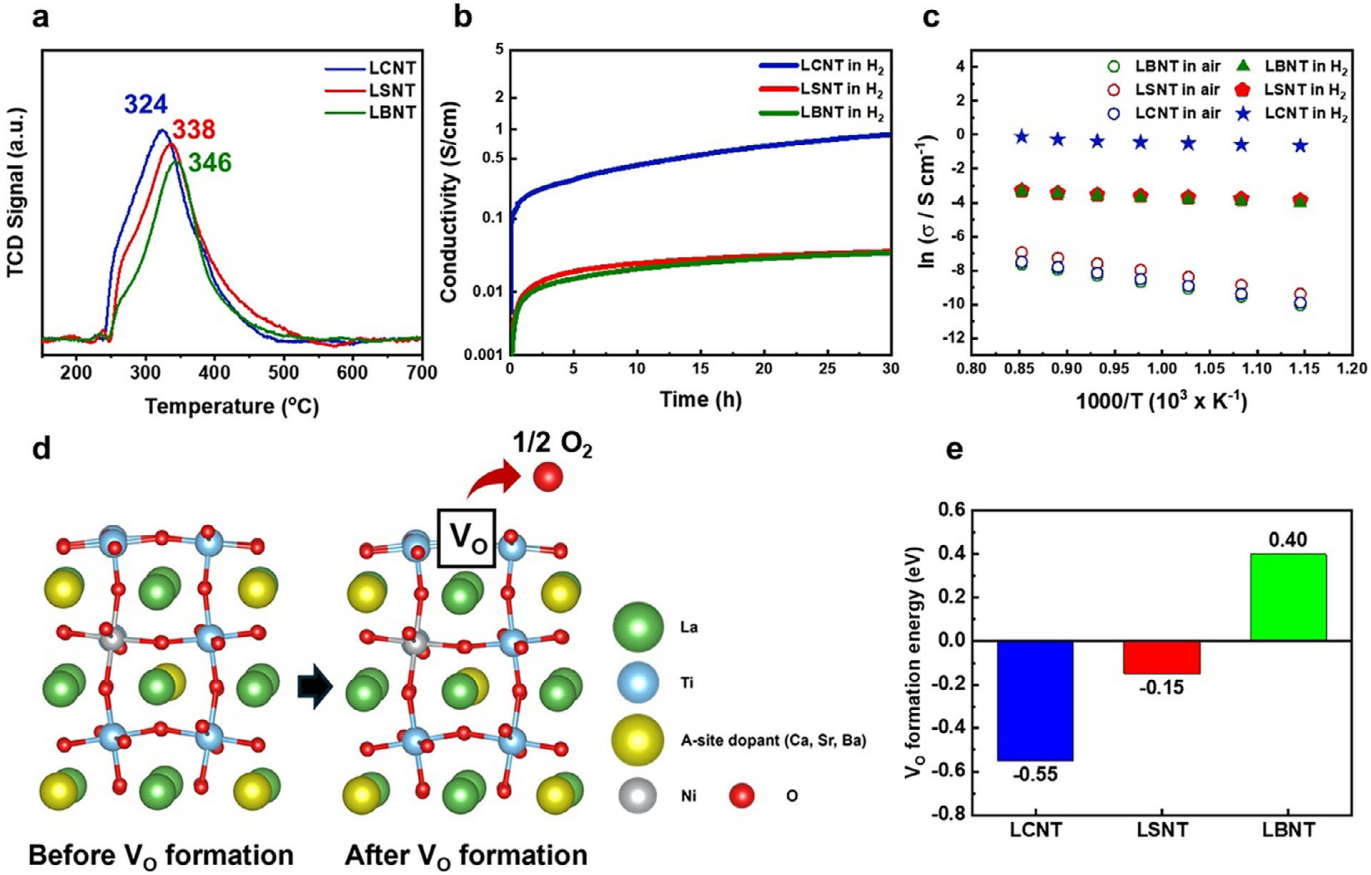
Reduction behavior of perovskite oxides. (a) H_2_‐TPR profiles of LCNT, LSNT, and LBNT. (b) Evolution of electrical conductivity during reduction in pure H_2_ at 900°C. (c) Arrhenius plot of electrical conductivity in air and pure H_2_. (d) Schematic illustration of the surface slab model used to calculate the oxygen vacancy formation energy. (e) Oxygen vacancy formation energies of perovskite oxides with different cation dopants.

To verify the effect of lattice distortion on reducibility, H_2_‐temperature‐programmed reduction (H_2_‐TPR) experiments were conducted (Figure 4a). A broad reduction peak with a shoulder was observed in the range of 200°C–450°C, corresponding to the reduction of Ni species from their oxidized states [29, 30]. A comparison of surface morphologies after H_2_ reduction at 200 and 400°C confirms that these TPR peaks are closely associated with Ni reduction and subsequent exsolution (Figure S5). Notably, LCNT exhibits the lowest reduction peak temperature (324°C) compared to LSNT (338°C) and LBNT (346°C), indicating the earlier initiation of Ni exsolution and a lower reduction barrier. The enhanced reducibility of the Ca‐doped perovskite oxides originates from the lattice distortion‐induced weakening of cation‐oxygen bonding. Ca substitution introduces intrinsically weaker A─O bonding [31] compared to Sr and Ba and induces pronounced octahedral tilting, leading to an elongated B─O bond length in LCNT despite the smaller ionic radius of Ca^2+^ (Table S1). Such octahedral distortion weakens cation‐oxygen interactions in a tensile‐strain‐like manner, thereby lowering the energetic barrier for reduction and subsequent metal exsolution. Consequently, lattice distortion facilitates reduction and subsequent exsolution by weakening cation‐oxygen interactions, thereby contributing to a reduced exsolution temperature [22].

To further investigate the reduction behavior, electrical conductivity was measured as an indirect descriptor of reducibility in air and H_2_ atmospheres using a four‐probe method (Figure 4b,c). Under air conditions, LCNT, LSNT, and LBNT showed very low conductivities (∼10^−4^ S cm^−1^), indicating electronic insulators. Upon reduction in an H_2_ environment at 900°C, the total conductivity of all the perovskite oxides increased by 2–4 orders of magnitude due to oxygen vacancy ($V_{O}^{2+}$) formation and partial reduction of Ti cations [32]. Notably, LCNT showed a substantially larger conductivity enhancement than LSNT and LBNT (Figure 4b). After H_2_ reduction at 900°C for 30 h, orthorhombic LCNT exhibited significantly higher electrical conductivity (0.52–0.90 S cm^−1^) than tetragonal LSNT (0.021–0.037 S cm^−1^) and LBNT (0.018–0.036 S cm^−1^) at 700–900°C. This increase in conductivity provides indirect but strong evidence for an increased bulk oxygen vacancy concentration in LCNT.

At the atomic scale, DFT calculations were performed to evaluate the oxygen vacancy formation energy, which serves as a descriptor of reducibility [33]. The calculations were carried out by introducing a single oxygen vacancy into the topmost layer of the perovskite surface [34]. The (100) facet, selected for its low surface energy, was employed for all models [35, 36, 37], and a Ti‐terminated surface was adopted based on its superior stability compared to Ni‐ or Ni/Ti‐co‐terminated surfaces (Figure 4d). Figure 4e displays the calculated oxygen vacancy formation energy of LCNT, LSNT, and LBNT. The oxygen vacancy formation energy of LCNT was found to be −0.55 eV, which is significantly lower than those of LSNT (−0.15 eV) and LBNT (0.40 eV). This lower oxygen vacancy formation energy is attributed to the structural distortion induced by Ca^2+^ doping, which facilitates the formation of oxygen vacancies. This computational result indicates the higher reducibility of LCNT, corresponding with experimental observations. The trend in oxygen vacancy formation energy, following the order LCNT < LSNT < LBNT, correlates well with the temperatures of the main TPR peaks of Ni. These DFT results provide an atomic‐scale origin for the experimentally observed enhanced reducibility of LCNT, demonstrating that lattice distortion intrinsically lowers the energetic barrier for oxygen vacancy formation and thereby drives metal exsolution in perovskite oxides.

To experimentally validate the oxygen vacancy formation tendency suggested by DFT calculations, X‐ray photoelectron spectroscopy (XPS) analysis of the O 1s spectra was conducted (Figure S6). The O 1s peaks were deconvoluted into three peaks of hydroxyl species (O_ad_, ∼532 eV), oxygen defects (O_defect_, ∼530.3 eV), and lattice oxygen (O_lattice_, ∼528.6 eV), where the O_defect_ is commonly associated with the oxygen vacancies in oxide systems [38]. Thus, the ratio of O_defect_/O_lattice_ is commonly used as a semi‐quantitative indicator of the relative concentration of oxygen vacancies [39]. In the as‐synthesized state, LCNT exhibited a higher O_defect_/O_lattice_ ratio of 0.61 compared to 0.46 for LSNT and 0.40 for LBNT, indicating a higher intrinsic oxygen vacancy concentration. This observation is consistent with the lower oxygen vacancy formation energy calculated for LCNT. After reduction at a high temperature of 900°C, the O_defect_/O_lattice_ ratio in all samples increased drastically to values of ∼10, indicating the extensive formation of oxygen vacancies. Despite this large increase in absolute vacancy concentration, the differences among the oxides became less pronounced due to rapid surface saturation of oxygen vacancies. As a result, the surface‐sensitive nature of XPS reflects the saturated surface state rather than the intrinsic bulk defect properties. These results demonstrate that lattice distortion in LCNT intrinsically promotes oxygen vacancy formation. While XPS captures the intrinsic trend in the as‐synthesized state, the enhanced electrical conductivity provides complementary evidence for increased bulk oxygen vacancy concentration under reducing conditions, consistent with both the DFT calculations and experimental observations.

**FIGURE 5:**
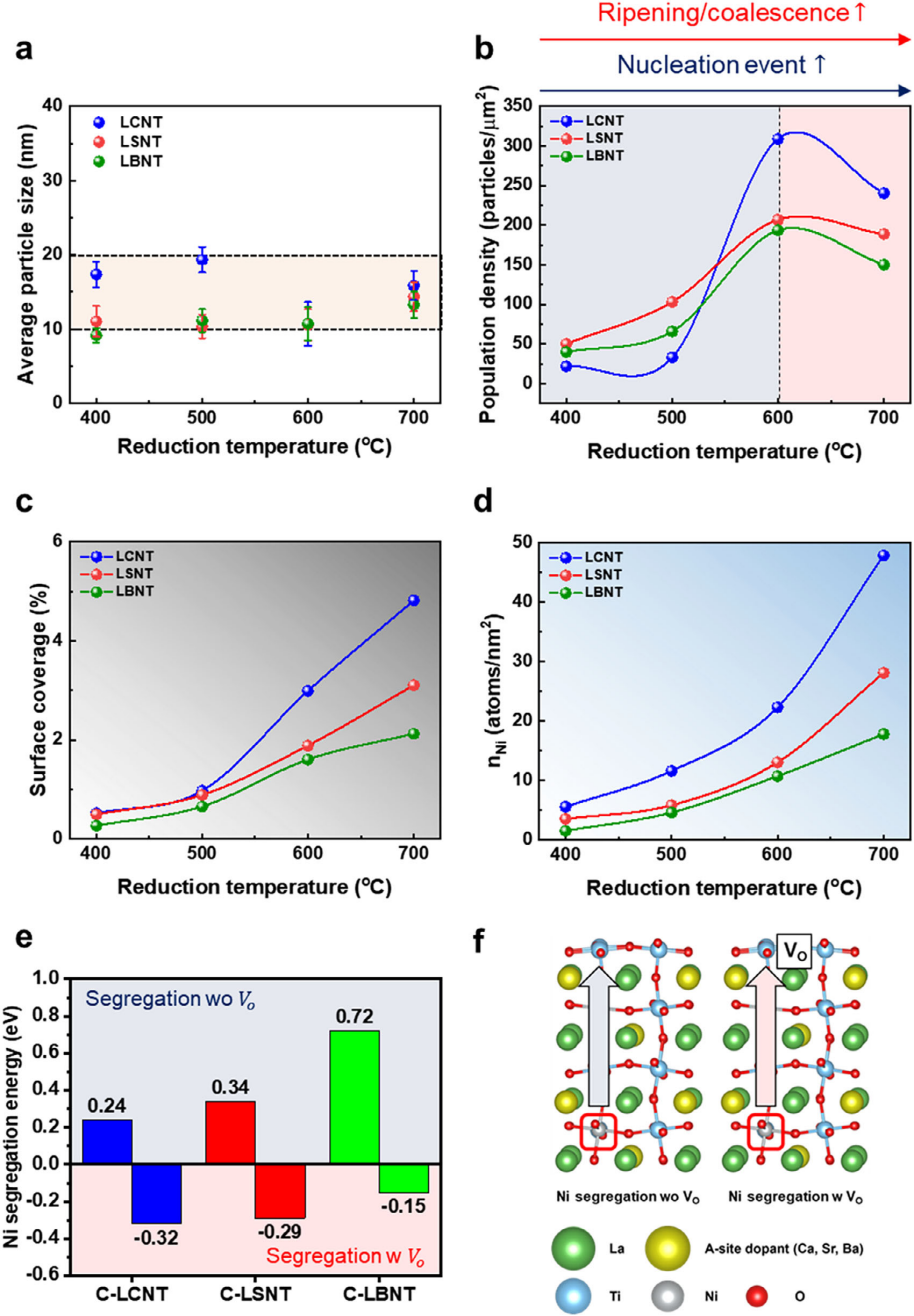
Exsolution tendency of perovskite oxides. (a) Average particle size, (b) population density, (c) surface coverage, and (d) extent of surface‐exsolved Ni of exsolved nanoparticles on the perovskite oxides reduced at 400–700°C for 20 h. (e) Ni segregation energies of computational perovskite oxides with and without surface oxygen vacancy. (f) Schematic illustration of the surface slab model used to calculate the Ni segregation energy.

To verify the exsolution of Ni nanoparticles, oxide pellets of the perovskites were reduced in H_2_ at 400–700°C for 20 h then their surface morphologies were explored. To minimize the influence of grain boundary effects, the central regions of the perovskite oxide grains (grain sizes of 1–5 µm) were selectively examined. The surfaces of the pristine oxides were clean and smooth with no visible secondary phases, whereas after reduction at all temperatures, uniformly distributed nanoparticles were observed on the oxide surfaces, corresponding with the XRD results (Figures S7 and S8). The exsolution behavior was quantitatively analyzed by evaluating the particle size, population density, surface coverage, and the number of exsolved atoms (Figure 5a–d). The exsolved nanoparticles exhibited a size range of 9–20 nm across all reduction temperatures (400–700°C), with low standard deviations (< 3 nm), indicating high size uniformity regardless of composition or temperature (Figure 5a). The population density increased with increasing reduction temperature, reaching maximum values of 193–309 particles µm^−2^ at 600°C for each sample (Figure 5b). This trend is consistent with classical nucleation theory, where higher temperatures promote nucleation rates [6]. However, at 700°C, the population density decreased slightly, which can be attributed to accelerated particle ripening or coalescence at elevated temperatures, as reported in previous ex situ [40, 41] and in situ studies [42, 43].

Among the perovskite oxides, LCNT exhibited a higher population density of exsolved nanoparticles (240–309 particles µm^−2^) at elevated reduction temperatures (600–700°C) compared to LSNT (189–207 particles µm^−2^) and LBNT (150–193 particles µm^−2^). Although reduction at 600°C yielded the highest population density for all the perovskite oxides, the surface coverage continued to increase with further temperature elevation (Figure 5c). This trend indicates that higher reduction temperatures are crucial for increasing the catalytically active surface area. The exsolution behavior of LCNT demonstrated the most favorable catalytic characteristics, featuring higher particle densities and greater surface coverage under the reduction conditions tested. This enhanced exsolution behavior of LCNT at elevated temperatures is likely driven by a greater extent of Ni exsolution facilitated by lattice distortion, in agreement with the phase analysis results (Figures 3f and 5d).

To elucidate the exsolution behavior from a theoretical perspective, the segregation energy for Ni migration from the bulk to the topmost surface layer was evaluated. The segregation energy serves as a descriptor of the exsolution tendency of B‐site cations, where lower values indicate a stronger driving force for surface segregation [44, 45]. A lower segregation energy implies a stronger tendency for the cations to migrate toward the surface. Segregation energies were first calculated in the absence of surface oxygen vacancies to investigate the intrinsic tendency of Ni migration prior to reduction, and subsequently in the presence of oxygen vacancies to capture the reduction‐induced exsolution behavior (Figure 5e,f). In the absence of oxygen vacancies, all compositions exhibited positive segregation energies, indicating that Ni segregation is energetically unfavorable before reduction. Notably, LCNT showed a lower segregation energy of 0.24 eV compared to 0.34 eV for LSNT and 0.72 eV for LBNT, suggesting a reduced energy barrier for Ni segregation. Under reduction conditions with surface oxygen vacancies, LCNT has exhibited the most negative segregation energy (−0.32 eV), compared to −0.29 eV for LSNT and −0.15 eV for LBNT, indicating the strongest thermodynamic driving force for Ni segregation. These trends are consistent with the experimental observations of enhanced Ni exsolution, larger particle sizes, and higher particle densities in LCNT.

### 2.3 Electrochemical Catalysis

**FIGURE 6:**
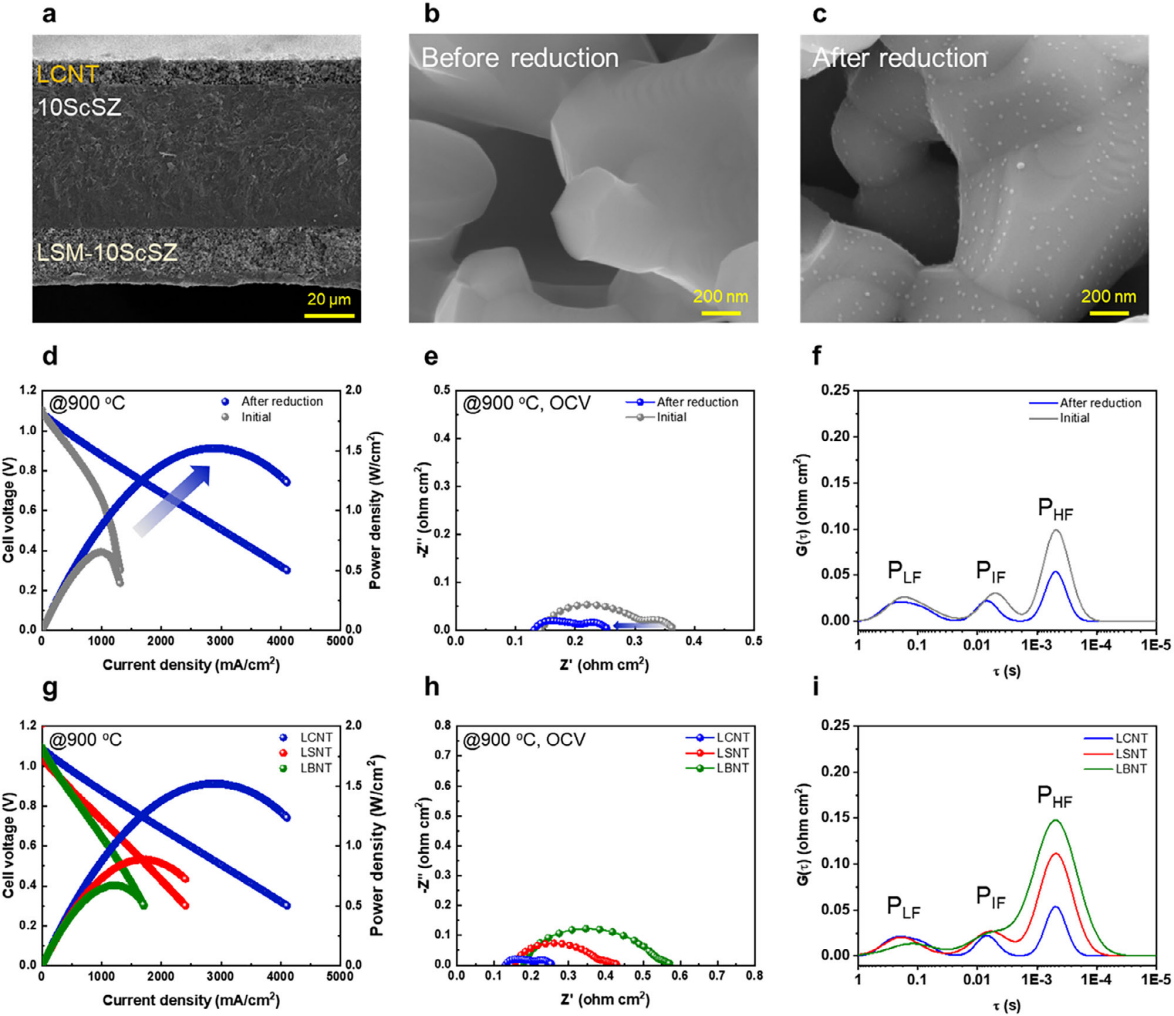
Effect of reduction on electrochemical performance. (a) Cross‐sectional microstructure of the single cell with an LCNT electrode. Surface morphologies of LCNT electrode (b) before and (c) after H_2_ reduction. (d) *I‐V‐P* curves, (e) EIS spectra, and (f) DRT analysis of LCNT‐based cells. Comparison of LCNT, LSNT, and LBNT electrodes in (g) *I‐V‐‐P* curves, (h) EIS spectra, and (i) DRT results.

The perovskite oxides were employed as fuel electrodes in an electrolyte‐supported SOFC configuration with a conventional electrolyte and air electrode, specifically LCNT (∼10 µm) / (Sc_2_O_3_)_0.10_(CeO_2_)_0.01_(ZrO_2_)_0.89_ (10ScSZ, ∼60 µm) / (La_0.8_Sr_0.2_)_0.95_MnO_3_ (LSM)‐10ScSZ (∼20 µm), to evaluate their electrochemical activity (Figure 6a). As shown in Figure 6b, the surface of the pristine perovskite electrode scaffold was clean and smooth, without any impurities. After reduction in H_2_ fuel, nanoparticles were exsolved on the electrode surface (Figure 6c). The electrochemical performance of the perovskite oxide electrodes can be enhanced by nanoparticle exsolution and oxygen vacancy formation during H_2_ reduction, which increase the density of catalytically active sites and improve charge transport properties [9]. To verify the effect of the reduction and exsolution, cell performance before and after reduction was compared using current‐voltage‐power (*I*‐*V*‐*P*) characterization and electrochemical impedance spectroscopy (EIS). While the initial cell with an LCNT electrode showed a maximum power density (MPD) of 0.65 W cm^−2^ at 900°C, the fully‐reduced cell exhibited a markedly higher MPD of 1.52 W cm^−2^ at 900°C, corresponding to a 2.3‐fold enhancement (Figure 6d). This improvement in MPD is attributed to the decrease in both the ohmic resistance (R_o_) and polarization resistance (R_p_), resulting from enhanced electrical conductivity and the generation of electrochemically active sites after reduction (Figure 6e). In particular, the exsolved nanoparticles provide metallic active sites for H_2_ adsorption/dissociation and extend the triple‐phase boundaries (TPBs), thereby facilitating surface reactions and charge‐transfer processes at the fuel electrode. Consequently, R_p_ decreased significantly from 0.220 to 0.121 Ω cm^2^ at 900°C, whereas R_o_ exhibited only a minor reduction from 0.142 to 0.132 Ω cm^2^ at 900°C. This indicates that the performance enhancement is predominantly governed by improved electrode kinetics rather than bulk ohmic losses.

To further elucidate the electrode polarization behavior, a distribution of relaxation times (DRT) calculation was performed (Figure 6f). Three characteristic peaks corresponding to physical and electrochemical processes were identified. Generally, peaks in the low‐ and intermediate‐frequency regions (P_LF_ and P_IF_) are associated with gas diffusion and adsorption/dissociation processes on the titanate/ferrite perovskite electrodes [46, 47, 48, 49]. The P_LF_ showed negligible change after reduction because gas‐diffusion‐related impedance is typically governed by microstructural parameters such as porosity and pore connectivity, which remained largely unchanged during reduction (Figure 6b,c). A slight decrease in the intensity of the P_IF_ is attributed to the enhanced H_2_ adsorption and dissociation behavior on the exsolved nanoparticles. In contrast, the high‐frequency peak (P_HF_), typically associated with faster charge‐transfer processes [50], showed a significant decrease in intensity after reduction, indicating a strong correlation with the enhanced electrochemical activity of the fuel electrode. These results imply that Ni exsolution and the formation of extended active sites (e.g., Ni‐oxide interfaces and oxygen vacancies) play a crucial role in accelerating charge‐transfer and reducing the electrode polarization resistance.

Single‐cell performances of the LCNT, LSNT, and LBNT electrodes were evaluated after full reduction using the same cell configuration and similar component thicknesses to compare their electrochemical activities. As discussed above, LCNT exhibits higher reducibility and more favorable exsolution behavior, resulting in higher particle densities and greater electrical conductivity compared to LSNT and LBNT (Figures 4c and 5). As a result, the LCNT‐based cell delivered the highest MPD of 1.52 W cm^−2^ at 900°C, outperforming the cells with LSNT (0.89 W cm^−2^) and LBNT (0.67 W cm^−2^) (Figure 6g). This superior performance originates from both a lower R_o_ of 0.132 Ω cm^2^ and a lower R_p_ of 0.121 Ω cm^2^ at 900°C compared to those of LSNT (R_o_: 0.158 Ω cm^2^, R_p_: 0.270 Ω cm^2^) and LBNT (R_o_: 0.179 Ω cm^2^, R_p_: 0.392 Ω cm^2^), despite having the same electrolyte and air electrode (Figure 6h). This highlights the dominant role of the fuel electrode in governing the overall electrochemical performance. DRT analysis further revealed that the P_LF_ and P_IF_ components showed minimal differences among the three cells, indicating that mass transport effects were comparable due to their similar microstructures. In contrast, the intensity of the P_HF_ followed the order LCNT < LSNT < LBNT, consistent with the observed trend in R_p_ (Figure 6i). This demonstrates that the perovskite composition strongly affects the charge‐transfer kinetics at the TPBs during the hydrogen oxidation reaction.

The high electrochemical activity of the LCNT electrode can be explained by its higher density of extended active sites resulting from enhanced reducibility and dynamic exsolution behavior (Figure S9). Although the exsolved nanoparticles showed similar average sizes of 17.9 ± 1.6 nm (LCNT), 17.2 ± 1.1 nm (LSNT), and 17.1 ± 3.0 nm (LBNT), the LCNT electrode displayed a notably higher population density (154.9 ± 36.8 particles µm^−2^) than LSNT (133.1 ± 28.8 particles µm^−2^) and LBNT (79.4 ± 29.3 particles µm^−2^), reflecting a higher extent of Ni exsolution. This suggests that the increased amount of exsolved Ni in LCNT is distributed over a larger number of nucleation sites, likely associated with an enhanced segregation tendency under lattice distortion, thereby maintaining a comparable particle size despite a significantly higher particle population. As a result, LCNT exhibited a higher extended TPB length density (8542 ± 1191 nm nm^−2^) compared to LSNT (7174 ± 1394 nm nm^−2^) and LBNT (4065 ± 985 nm nm^−2^). Because TPB density and catalyst surface area directly determine the availability of electrochemical reaction sites, these structural advantages explain the superior charge‐transfer activity of LCNT. These results demonstrate that lattice distortion‐enhanced reducibility and dynamic exsolution play a pivotal role in boosting the electrochemical performance by increasing the density of active sites.

### 2.4 Thermochemical Catalysis

Dry reforming of methane (DRM) converts greenhouse gases (CO_2_, CH_4_) into useful syngas (H_2_, CO) via CO_2_ + CH_4_ ↔ 2CO + 2H_2_, providing a relevant reaction platform for evaluating catalytic performance under SOFC‐compatible fuel conditions [51, 52, 53, 54, 55]. Before the thermochemical DRM test, fine submicron‐sized powders of the perovskite oxides were reduced in situ under an H_2_ environment at 800°C for 20 h in a fixed‐bed reactor, yielding Ni@LCNT, Ni@LSNT, and Ni@LBNT (Figure S10). Metallic Ni nanoparticles were generated from the oxide supports during reduction, forming abundant catalytically active sites, such as exposed Ni surfaces and Ni‐oxide interfaces (Figure S11a–c). While the average sizes of the exsolved Ni nanoparticles on the perovskites were comparable, Ni@LCNT exhibited a higher particle population density of 97.6 particles µm^−^^2^ than Ni@LSNT (75.3 particles µm^−^^2^) and Ni@LBNT (27.9 particles µm^−^^2^) (Figure S11d–i). Although the trends in particle size and population density were comparable to those observed on flat bulk or porous scaffold surfaces, the absolute population densities were lower, likely due to kinetic limitations associated with the submicron‐sized powders, such as restricted ion diffusion and limited reactant availability [56]. Notably, all reduced catalysts exhibited robust socketed Ni particles, providing high resistance both to agglomeration at elevated temperatures and to detachment induced by carbon deposition [9, 10, 57].

**FIGURE 7:**
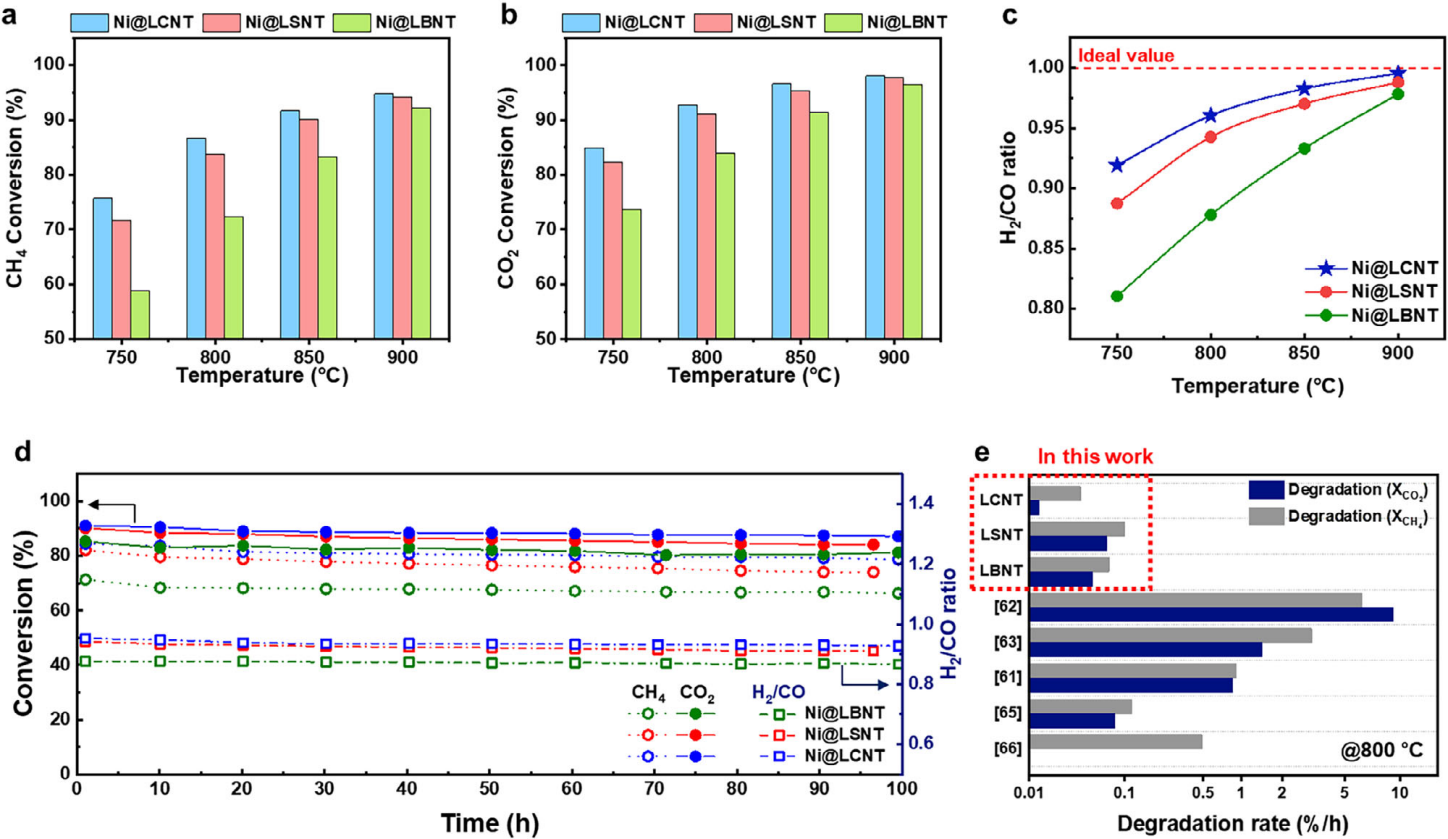
Catalytic performance of Ni‐exsolved perovskite oxides in DRM. (a) CH_4_ conversion, (b) CO_2_ conversion, and (c) H_2_/CO ratio of exsolution catalysts at different reaction temperatures. (d) CH_4_/CO_2_ conversion and H_2_/CO ratio during continuous reaction at 800°C for 100 h (CH_4_:CO_2_ = 1:1, GHSV = 30000 mL g^−1^ h^−1^). (e) Comparison of the degradation rates of Ni‐containing catalysts under the DRM process at 800°C.

To evaluate the DRM catalytic performance of the catalysts, CH_4_ and CO_2_ conversion activities and selectivity (H_2_/CO) were measured under undiluted gas conditions at 750–900°C (50 mL min^−1^, CH_4_:CO_2_ = 1:1, gas hourly space velocity, GHSV = 30 000 mL g^−1^ h^−1^) (Figure 7a–c). For all the catalysts, higher temperatures promoted CH_4_ and CO_2_ conversions due to the endothermic nature of the DRM reaction. Notably, Ni@LCNT exhibited the highest conversion activities over the entire temperature range. This superior performance is attributed to its higher extent of Ni exsolution and greater particle population density, providing a high density of active sites for CH_4_ dehydrogenation and CO_2_ dissociation. Indeed, Ni@LCNT showed significantly higher CH_4_ and CO_2_ conversions than pristine LCNT, highlighting the critical role of exsolved Ni nanoparticles in DRM catalysis (Figure S12). The CO_2_ conversion exceeded that of CH_4_ for all catalysts due to side reactions such as the reverse water‐gas shift reaction (CO_2_ + H_2_ → CO + H_2_O), which lowers the H_2_/CO ratio from the ideal value of 1.0. Ni@LCNT consistently exhibited higher H_2_/CO ratios than Ni@LSNT and Ni@LBNT, indicating superior selectivity toward the DRM pathway.

To elucidate the support effects, the surface properties of the perovskite catalysts were examined. The specific surface areas (SSAs) of the as‐prepared powders prior to exsolution were measured by N_2_ adsorption‐desorption isotherms (Figure S13a). The Brunauer‐Emmett‐Teller (BET) surface area of LCNT, LSNT, and LBNT were 6.28, 5.84, and 5.90 m^2^/g, respectively, corresponding to a submicron range with estimated particle radii of 83–87 nm (Figure S13b). The similar SSAs reflect identical synthesis and milling procedures, indicating that differences in catalytic performance primarily arise from variations in the exsolved Ni active sites rather than surface area effects. Thus, the surface acidity and basicity of the catalysts were further investigated using NH_3_‐ and CO_2_‐temperature programmed desorption (TPD) (Figure S14). NH_3_‐TPD profiles revealed only weak acidic sites (∼100°C), with no medium or strong acidic sites detected at higher temperatures [58]. The absence of strong acidity suppresses the formation of inactive carbon species, contributing to catalyst stability [59]. In contrast, CO_2_‐TPD demonstrated that Ni@LCNT possessed both weak (∼100°C) and strong (∼750°C) basic sites, whereas Ni@LSNT and Ni@LBNT exhibited only weak basicity. The strong basic sites in Ni@LCNT, originating from Ca‐containing oxide species, promote CO_2_ adsorption and activation, facilitating CH_4_ reforming and the oxidation of deposited carbon [60, 61]. This enhanced surface basicity, together with the socketed Ni structure, synergistically improves catalyst durability. Consistent with these findings, Ni@LCNT demonstrated superior stability during continuous DRM operation at 800°C for 100 h, exhibiting the lowest degradation rates in both CH_4_ and CO_2_ conversion compared to Ni@LSNT, Ni@LBNT, and previously reported Ni nanoparticles‐based catalysts of Ni@Ba_8_Ta_6_NiO_24_ [62], Ni@Hol S1 zeolite [63], Ni@SiO_2_ [64], Ni@Al_2_O_3_ [65], Ni@Mg‐Al layered double hydroxide [66] (Figure 7d,e). These results demonstrate that Ni‐─exsolved LCNT delivers outstanding DRM performance combining high activity, selectivity, and long‐term stability, outperforming previously reported Ni‐based catalysts (Table S2). This superior performance is attributed to (1) the uniform distribution and robust socketed structure of the exsolved Ni nanoparticles, which enhance resistance to sintering and detachment while suppressing carbon growth, (2) the high extent of Ni exsolution facilitated by lattice distortion, leading to an increased density of active sites for CH_4_ activation, and (3) the strong basic nature, which promotes CO_2_ adsorption and activation and facilitates the removal of carbon species. These factors synergistically contribute to improved catalytic performance by simultaneously enhancing reactant activation and inhibiting catalyst deactivation.

## 3 Conclusion

The lattice distortion‐enhanced Ni exsolution on the designed titanate perovskite oxides was investigated to achieve high catalytic performance in energy conversion. Substitution of smaller A‐site cations, such as Ca^2+^, in the perovskite oxides induces a higher degree of octahedral tilting than that of larger cations (La^3+^, Sr^2+^ and Ba^2+^). Such lattice distortion increased thermodynamic instability, weakened cations‐oxygen bonds, and thereby promoted oxygen vacancy formation, ultimately facilitating Ni segregation. During high‐temperature reduction, the lattice distortion in the perovskite oxides gradually relaxed, indicating that it acts as a transient yet effective driving force for exsolution. Consequently, the Ca^2+^‐doped perovskite oxides with greater lattice distortion exhibited more dynamic exsolution behavior, characterized by higher particle population density and greater surface coverage compared to the Sr^2+^‐ or Ba^2+^‐doped perovskite oxides. The enhanced exsolution of the socketed nanoparticles significantly increased the number of active sites for hydrogen oxidation reactions as well as for CH_4_ and CO_2_ dissociation, achieving high activity in both electrochemical and thermochemical processes. This lattice distortion‐driven strategy offers an effective approach for inducing nanoparticle exsolution in a broad range of material systems, thereby advancing the design of high‐performance catalysts for energy conversion applications.

## 4 Methods

### 4.1 Sample Preparation

Perovskite oxides were synthesized via a solid‐state reaction method. The starting materials were La_2_O_3_ (99.9%, Alfa Aesar), CaCO_3_ (99.5%, Thermo Scientific), SrCO_3_ (99.9%, Sigma–Aldrich), BaCO_3_ (99%, Thermo Scientific), Ni(NO_3_)_2_·6H_2_O (99%, Acros Organics) and TiO_2_ (99.8%, Sigma–Aldrich). The oxide and carbonate powders were calcined overnight at 300°C to remove moisture and impurities from the surface. The calcined powders and nitrate reagent with designed stoichiometric compositions were stirred in a beaker using deionized (D.I.) water and a magnetic stir bar for 24 h. After drying in an oven at 70°C, the mixtures were ground using an agate mortar and pestle, then filtered through a 70‐mesh sieve (opening diameter ≈ 200 µm). The powders were compacted into pellets and subjected to pre‐sintering in a furnace at a temperature of 1000°C for 3 h. This pre‐sintering step was performed to facilitate the diffusion of elements and promote the formation of intermediate phases. The pre‐sintered pellets were ground using an agate mortar and pestle. After sieving, approximately 3 g of the powder was shaped into a pellet and sintered at 1300°C for 3 h to achieve the perovskite phase. The synthesized perovskite oxides were then crushed in an alumina jar with ethanol as a milling medium using a planetary ball mill at 300 rpm for 4 h to obtain fine powders for analysis and catalytic reactions.

### 4.2 Characterization

The crystal structures and phases of the materials were investigated using powder XRD (Rigaku SmartLab, Cu K_α_ radiation) in the 2θ range of 20°–90° with a step of 0.02°. The XRD patterns were refined by Rietveld analysis using GSAS and VESTA software programs to obtain the crystal structures, cell parameters, and phase fractions. To compare the cell parameters of the perovskite oxides with different space groups, the pseudocubic cell parameter (*a_p_*) was calculated from the cell volume (*V*_*unit* *cell*_) and the number of ABO_3_ in a unit cell ($n_{ABO_{3}}$).

(1) $$a_{p}={\left(\frac{V_{unit\;cell}}{n_{ABO_{3}}}\right)}^{\frac{1}{3}}$$

The molar ratio of Ni to the perovskite phase in the materials was calculated from the phase fractions obtained from the refined information. The mean A─O, B─O bond lengths and B—O—B angles were extracted from the refined data using VESTA, followed by estimation of the τ of perovskite oxides.

(2) $$\tau =\frac{r_{A}+r_{O}}{\sqrt{2}\left(r_{B}+r_{O}\right)}$$

where *r*_A_, *r*_B_, and *r*_O_ are the ionic radii of the A‐site cation, B‐site cation, and oxygen anion, respectively.

XPS (Thermo Fisher NEXSA G2) was performed to estimate the oxidation states of O on the surface of perovskite oxides before and after reduction. The exsolved nanoparticles on the surface were characterized using a scanning electron microscope (JEOL 7001F) and a field emission transmission electron microscope (Thermo Fisher Scientific Talos F200X) with an energy dispersive spectrometer (EDS). Quantitative analyses of the nanoparticle size, volume (*V_particle_*), and population density of the exsolved particles were conducted using the ImageJ software under the assumption that both the oxide particles and the exsolved nanoparticles are spherical and that the pellet surface is flat. The amount of exsolved Ni atoms (*n_Ni_*) per oxide surface area was estimated from the total volume of the exsolved particles, Avogadro's number (*N_A_* = 6.022 × 10^23^ atoms mol^−1^), the Ni density (*ρ* = 8.907 g cm^−3^), the Ni molar mass (*M_Ni_* = 58.6934 g mol^−1^), and the surface area (A).

(3) $$n_{Ni}=\frac{\rho \frac{N_{A}}{M_{Ni}}\sum V_{particle}}{A}$$

The surface coverage (θ) was estimated from the circlar area of the exsolved nanoparticles (*A_particle_*).

(4) $$\theta \;\left(\%\right)=100\times \frac{\sum A_{particle}}{A}$$

The extended TPB length density (λ) on the surface of the perovskite electrodes was calculated from the circumferences of the exsolved Ni particles.

(5) $$\lambda =\frac{\sum \pi d}{A}$$

where *d* is the diameter of each exsolved Ni nanoparticle. The electrical conductivities of the perovskite oxides were measured at different temperatures in a quartz tube furnace under air and H_2_ environments using a DC 4‐probe technique. Dense and rectangular bar‐shaped samples (3 mm × 3 mm × 20 mm) for the conductivity measurements were prepared following the same solid‐state reaction method, including sintering at 1500°C for 6 h. H_2_‐TPR (AUTOCHEM II 2920, Micromeritics Instrument Corporation) was conducted to verify reducibility of the perovskite oxides. After heat treatment in a He environment at 300°C for 1 h, 200 mg of the powder samples were exposed to 10%H_2_/Ar and heated from 100 to 700°C. To evaluate the acidity/basicity of the catalysts, Ni‐exsolved perovskite oxides reduced at 800°C for 20 h were characterized by NH_3_‐ and CO_2_‐ TPD (AUTOCHEM II 2920, Micromeritics Instrument Corporation). The Ni‐exsolved catalysts were preheated in 10%H_2_/He at 800°C for 1 h, followed by treatment in a He atmosphere at 300°C for 1 h, to remove any oxidizing layers and impurities. NH_3_ and CO_2_ were adsorbed on the catalyst surfaces in 15%NH_3_/He and 15%CO_2_/He conditions at 50°C for 1 h, respectively. The gases were desorbed during heating in He from 50 to 900°C and detected using thermal conductivity detector (TCD). The specific surface area of as‐prepared oxide catalysts was measured by N_2_ sorption isotherm with BET analysis (BEL, BELSORP‐MINI II).

### 4.3 DFT Calculation

DFT calculations were performed using the Vienna Ab initio Simulation Package [67]. Exchange‐correlation energies were treated with the Perdew‐Burke‐Ernzerhof functional within the generalized gradient approximation. A kinetic energy cutoff of 400 eV was applied for plane wave expansion. For *k‐*point sampling of the Brillouin zone, a Monkhorst‐Pack grid of 3 × 3 × 3 was used for the bulk calculations and 3 × 3 × 1 for the slab models [68]. Gaussian smearing with a width of 0.05 eV was employed to determine the partial occupancies. Structural relaxations were carried out until the residual forces on all atoms were less than 0.03 eV/Å. To account for van der Waals interactions, Grimme's DFT‐D3 dispersion correction was applied. Additionally, to correct for the self‐interaction error associated with correlated d and f electrons, DFT‐D3+U calculations were conducted by introducing on‐site Hubbard U parameters: 7 eV for La 4*f* electrons, 5 eV for Ti 3*d* electrons, and 5.3 eV for Ni 3*d* electrons [69]. In the slab models, the bottom four atomic layers were fixed while the upper eight layers near the surface were fully relaxed, with a 15 Å vacuum layer added perpendicular to the surface.

The formation energy (*E_form_*) of perovskites was calculated according to the following equation (6):

(6) $$E_{\mathrm{form}}=E_{C-\mathrm{LMNT}}-E_{\mathrm{LTO}}-n\cdot \mu_{M}-n\cdot \mu_{\mathrm{Ni}}+n\cdot \mu_{\mathrm{La}}+n\cdot \mu_{\mathrm{Ti}}$$

where *E*_*C* *‐LMNT*_ and *E_LTO_* represent free energies of La_0.75_M_0.25_N_0.25_Ti_0.75_O_3_ (LMNT, M: Ca, Sr, Ba) and undoped LaTiO_3_. *µ*_*M*_, *µ*_*Ni*_, *µ*_*La*_, and *µ*_*Ti*_ are chemical potentials of M, Ni, La, and Ti, respectively. *n* represents the number of doped M and Ni atoms in the bulk modeling.

The oxygen vacancy formation energy ($E_{V_{O}\;formation}$) for perovskite oxides was calculated by introducing a single oxygen vacancy into the topmost layer according to equation (7):

(7) $$E_{V_{O}\;formation}=E_{V_{O}\;slab}-E_{slab}+\mu_{O}$$

where $E_{V_{O}\;slab}$ and *E_slab_* denote the total energies of the slab model with and without an oxygen vacancy, respectively, and µ_*O*_ represents the chemical potential of oxygen.

For the segregation energy (*E_seg_*) calculations, Ni migration was modeled from the seventh layer (counted from the top) to the topmost surface layer, and the corresponding energy change was evaluated as the segregation energy. The segregation energy is represented by equation (8):

(8) $$E_{seg}=E_{after\;seg}-E_{before\;seg}$$

where *E*_*after* *seg*_ and *E*_*before* *seg*_ represent the energies after and before segregation, respectively.

### 4.4 Cell Fabrication and Electrochemical Test

The 10ScSZ electrolyte supports were fabricated via a tape casting process. The tape casting slurry was prepared by mixing the ScSZ powder (DKKK, 10Sc1CeSZ) with an ethanol‐toluene medium, Triton‐X, polyvinyl butyral, and dibutyl phthalate using ball milling. The 10ScSZ tape was cast on a heating plate at 45°C using a doctor blade with a gap height of 400 µm. The green sheet was cut into 1‐inch circlar discs and sintered at 1400°C for 3 h. Fuel and air electrode inks were prepared by mixing the respective electrode powders, ethyl cellulose, and alpha‐terpineol using an agate mortar and pestle. The fuel electrode ink was screen‐printed over an area of 0.5 cm^2^ and sintered at 1200°C for 3 h. Subsequently, the air‐electrode ink of LSM (LSM20‐P, FCM)‐10ScSZ was printed and sintered at 1100°C for 3 h. Before the cell test, Pt paste was applied to both sides of the electrode as a current collector. The prepared cells were mounted on an alumina jig with Pt mesh and Pt wires and were sealed using glass sealant (Aremco‐Seal 617). For the fuel electrode, 200 sccm of H_2_ was humidified using a water bubbler, and 500 sccm of dry air was supplied to the air electrode via mass flow controllers. Cell tests were conducted using an electrochemical workstation (Biologics, VMP‐300). The *I* *‐V‐* *P* curves for each cell were measured by linear sweep voltammetry from the open circuit voltage (OCV) to 0.3 V, and EIS was performed at OCV over the frequency range of 200 kHz to 100 mHz. The DRT calculations were conducted using a Python‐based DRT tool applied to the EIS data.

### 4.5 Thermochemical Catalytic Test

DRM reactions were tested in a packed bed quartz reactor (inner diameter: 8 mm, length: 50 cm) within a tube furnace. 100 mg of the catalyst was loaded and fixed in the reactor using quartz wool and alumina balls. The inlet gases of CH_4_ and CO_2_ (1:1, Total flow: 50 mL min^−1^, GHSV: 30000 mL g^−1^ h^−1^) were injected into the reactor using mass flow controllers. The reacted outlet gases were analyzed using a gas chromatograph (Agilent 8890 GC system) with a carboxen‐1000 packed column and a TCD. Argon was used as the carrier gas at a constant flow rate of 23 mL min^−1^. The temperatures of both the injector and the TCD detector were maintained at a constant 140°C. The temperature of the column oven was maintained at 160°C for 10 min. The CH_4_/CO_2_ conversions (*X_G_*, G: CH_4_ or CO_2_) and the H_2_/CO ratio were calculated from the concentrations of the inlet and outlet gases.

(9) $$X_{CH_{4}}=\frac{F_{inlet}\times {\left[CH_{4}\right]}_{inlet}-F_{outlet}{\left[CH_{4}\right]}_{outlet}}{F_{inlet}\times {\left[CH_{4}\right]}_{inlet}}$$

(10) $$X_{CO_{2}}=\frac{F_{inlet}\times {\left[CO_{2}\right]}_{inlet}-F_{outlet}{\left[CO_{2}\right]}_{outlet}}{F_{inlet}\times {\left[CO_{2}\right]}_{inlet}}$$

(11) $$H_{2}/CO\;molar\;ratio={\left[H_{2}\right]}_{outlet}/{\left[CO\right]}_{outlet}$$

where [*CH*_4_], [*CO*_2_], [*H*_2_], and [*CO*] are the mole fractions of CH_4_, CO_2_, H_2_, and CO, respectively. *F_inlet_* and *F_outlet_* indicate the total gas flow rates of the inlet and outlet gases.

## Conflicts of Interest

The authors declare no conflicts of interest.

## Supporting information




**Supporting File**: advs75870‐sup‐0001‐SuppMat.docx.

## Data Availability

The data that support the findings of this study are available from the corresponding author upon reasonable request.
