# Supplementary material for: Lattice Distortion‐Driven Metal Exsolution in Perovskite Oxides
Source: Adv Sci (Weinh). 2026 Jun 1:e75870. Online ahead of print. doi: 10.1002/advs.75870 (PMC13335906; doi:10.1002/advs.75870)
Supplement: Supplementary file 1 — Supporting File: advs75870‐sup‐0001‐SuppMat.docx. [file ADVS-9999-e75870-s001.docx]

Supporting Information

**Lattice Distortion-driven Metal Exsolution in Perovskite Oxides**

*Yo Han Kim, Uchan Jeon, Hyeongwon Jeong, Bo-Ram Won, Jeong Woo Han* and Jae-ha Myung**

Y. H. Kim, H. Jeong, B.-R. Won, J.-ha Myung

Department of Materials Science and Engineering, Incheon National University, Incheon 22012, Republic of Korea.
*E-mail: [mjaeha@inu.ac.kr](mailto:mjaeha@inu.ac.kr) (J.-ha Myung)

U. Jeon, J. W. Han

Department of Materials Science and Engineering, Research Institute of Advanced Materials, Seoul National University, Seoul 08826, Republic of Korea.

*E-mail: [jwhan98@snu.ac.kr](mailto:jwhan98@snu.ac.kr) (J. W. Han)

Y. H. Kim and U. Jeon contributed equally to this work.

**
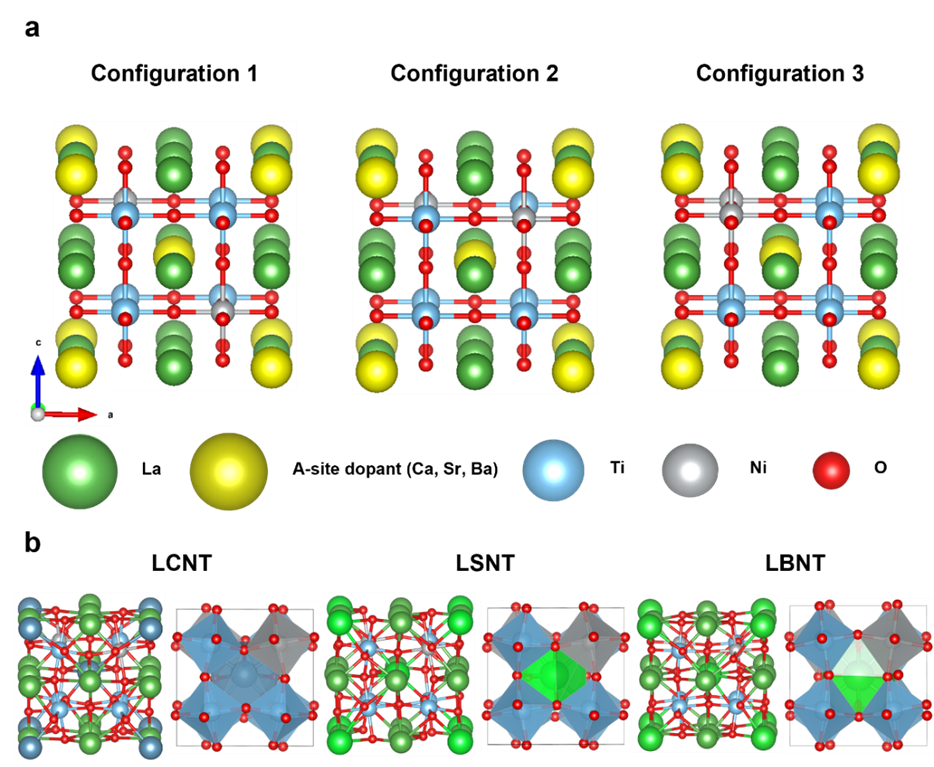
**

**Figure S1.** (a) Schematic representations of the considered doping configurations. The 2^nd^ configuration is found to be the most thermodynamically stable for all systems: LCNT, LSNT, and LBNT. (b) The fully optimized and tilted perovskite structures.

**
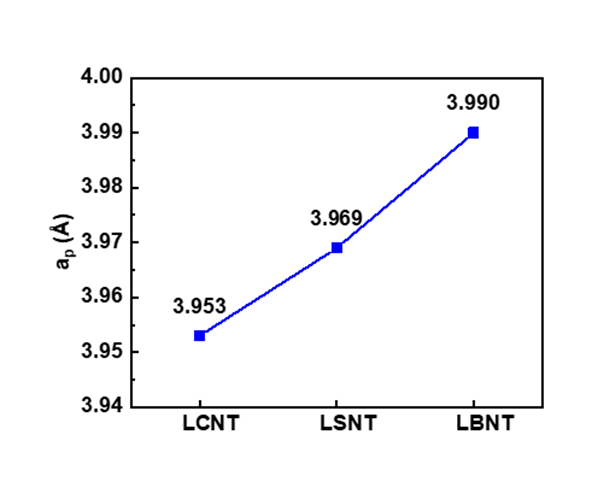
**

**Figure S2.** Pseudocubic cell parameter of LCNT, LSNT, and LBNT.


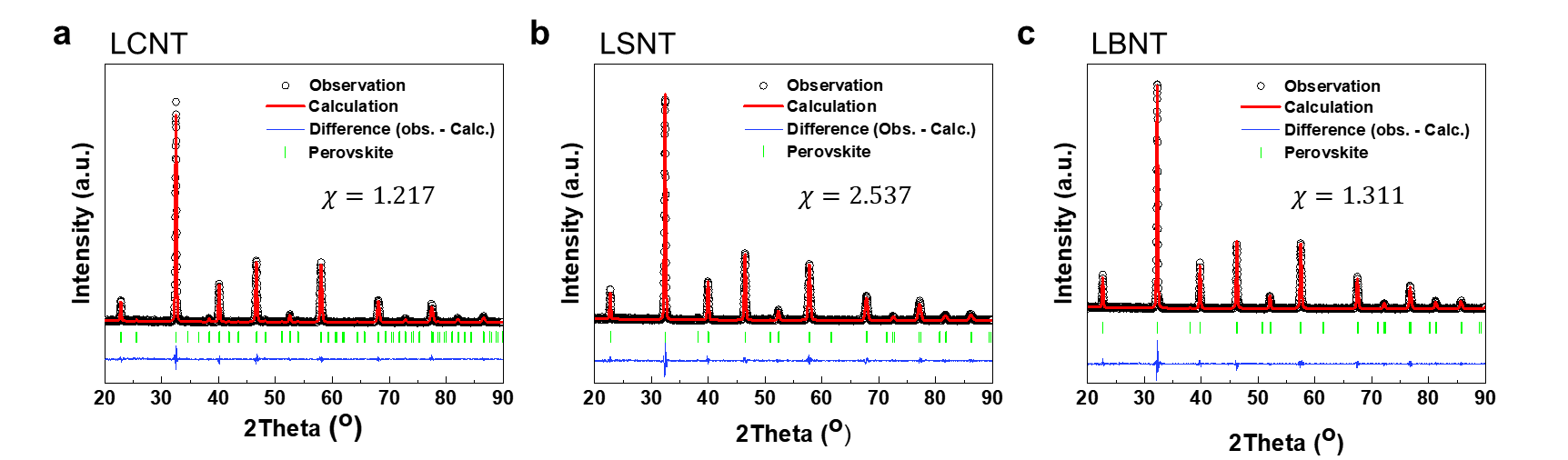


**Figure S3**. Refined XRD patterns of as-synthesized (a) LCNT, (b) LSNT, and (c) LBNT.


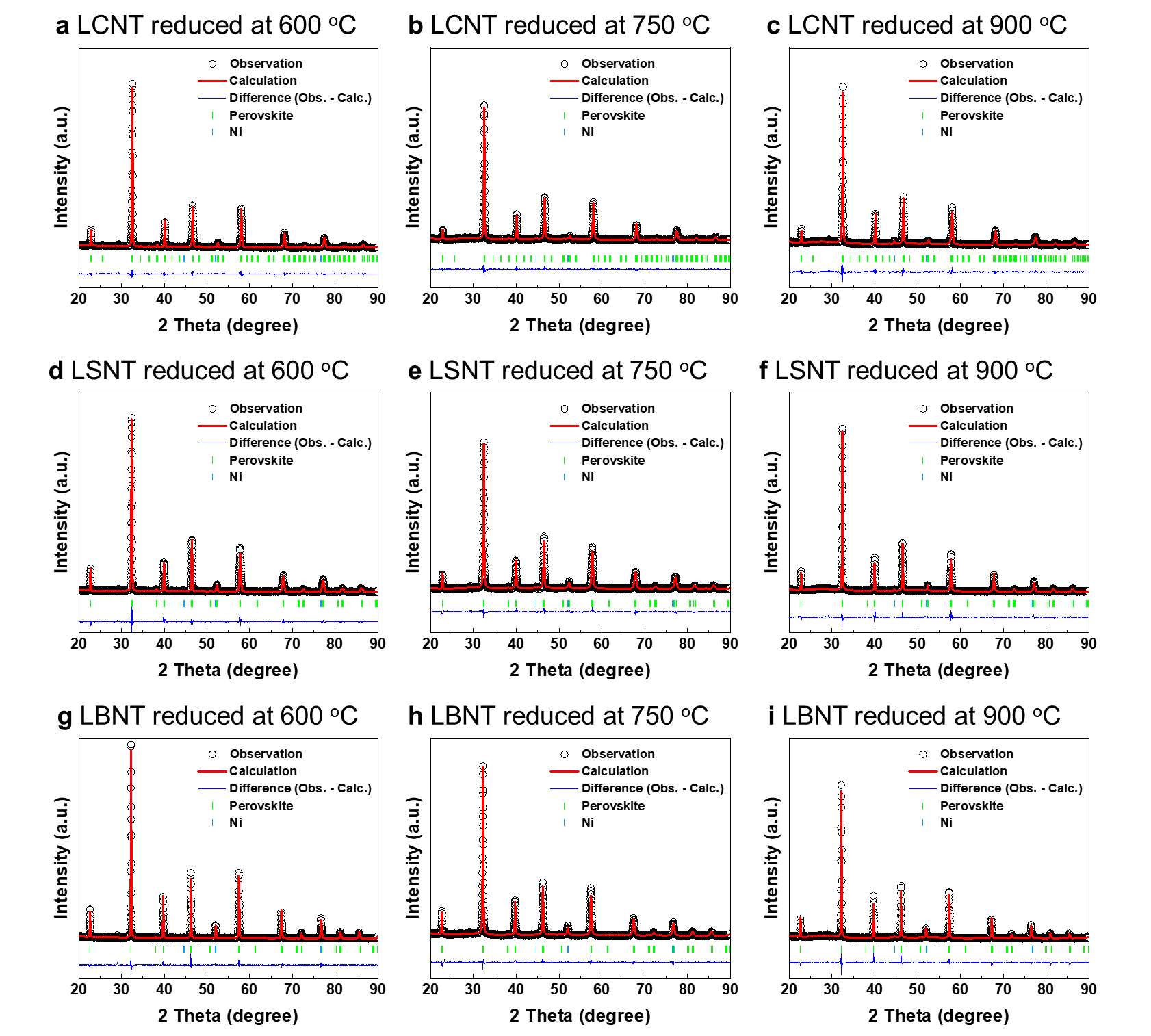


**Figure S4.** Refined XRD patterns of (a-c) LCNT, (d-f) LSNT, and (g-i) LBNT after reductions at 600, 750 and 900 °C for 20 h.

**
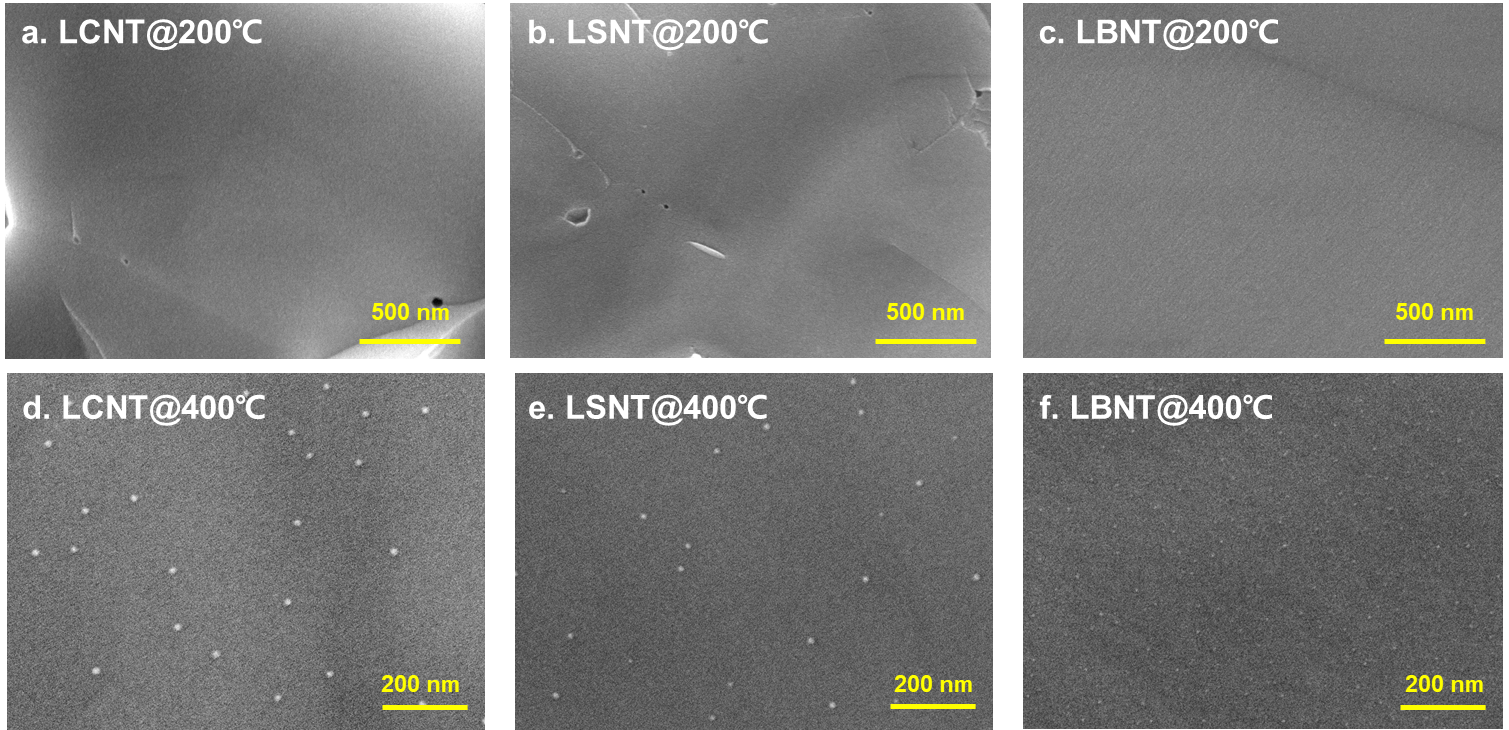
**

**Figure S5.** Surface morphologies of (a and d) LCNT, (b and e) LSNT, and (c and f) LBNT after reduction in pure H_2_ at 200 and 400 °C for 20 h.

**
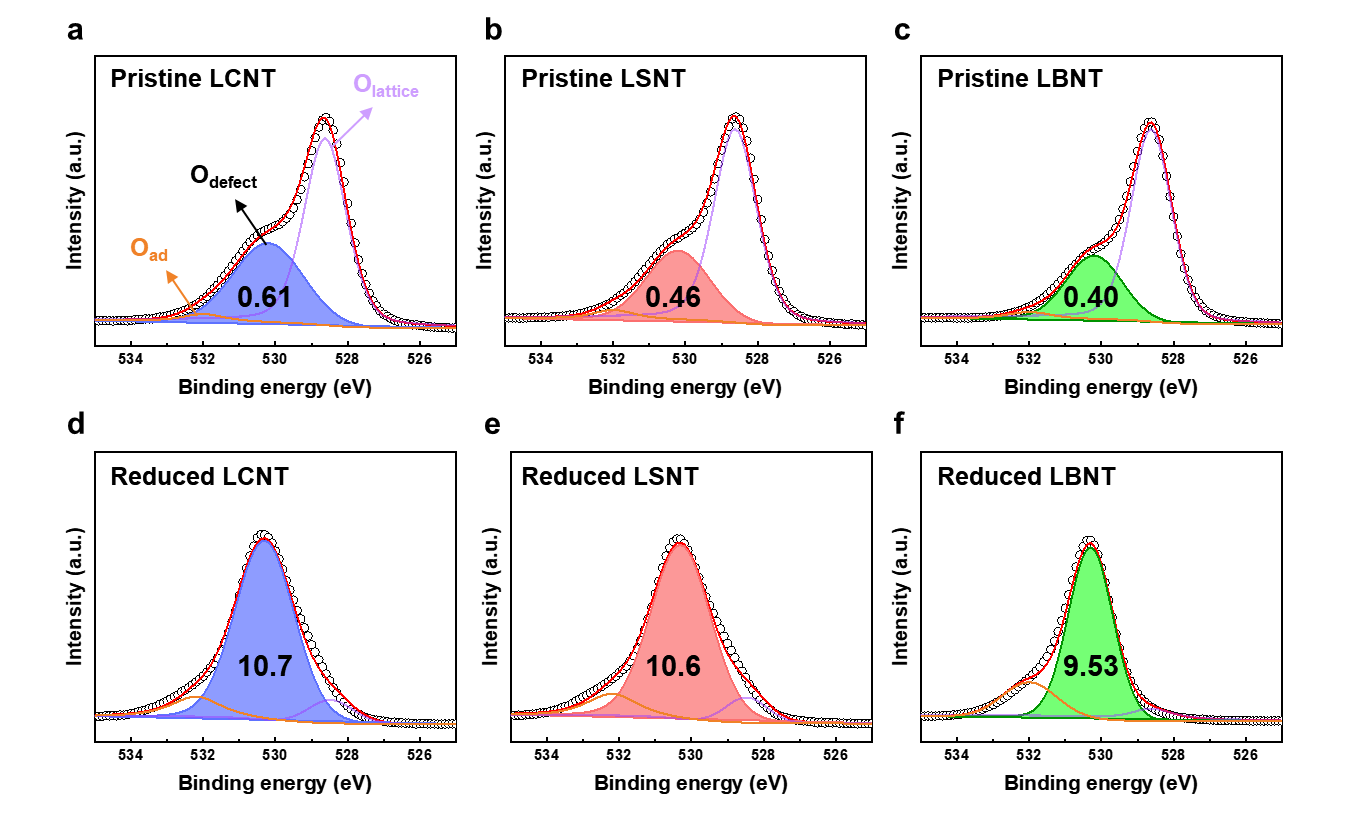
**

**Figure S6.** XPS O 1s spectra and corresponding O_defect_/O_lattice_ ratios of LCNT, LSNT, and LBNT measured (a–c) before and (d–f) after reduction at 900 °C for 12 h. The deconvoluted O 1s spectra consist of lattice oxygen (O_lattice_), defect-related oxygen (O_defect_), and adsorbed hydroxyl species (O_ad_).

**
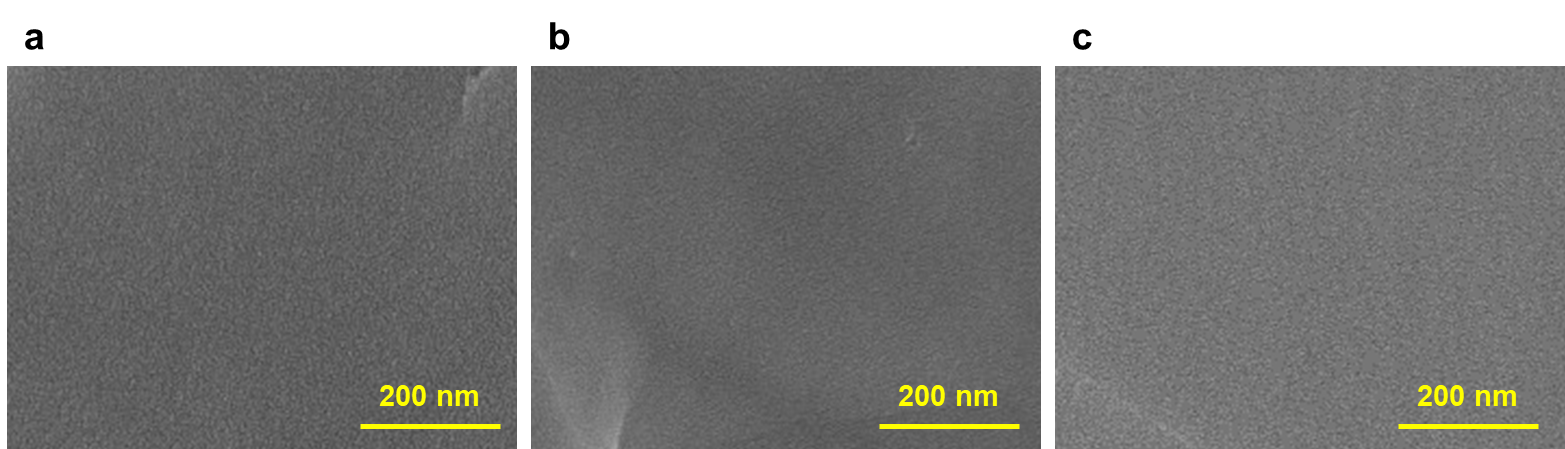
**

**Figure S7.** Surface morphologies of pristine (a) LCNT, (b) LSNT, and (c) LBNT synthesized by solid state reaction.


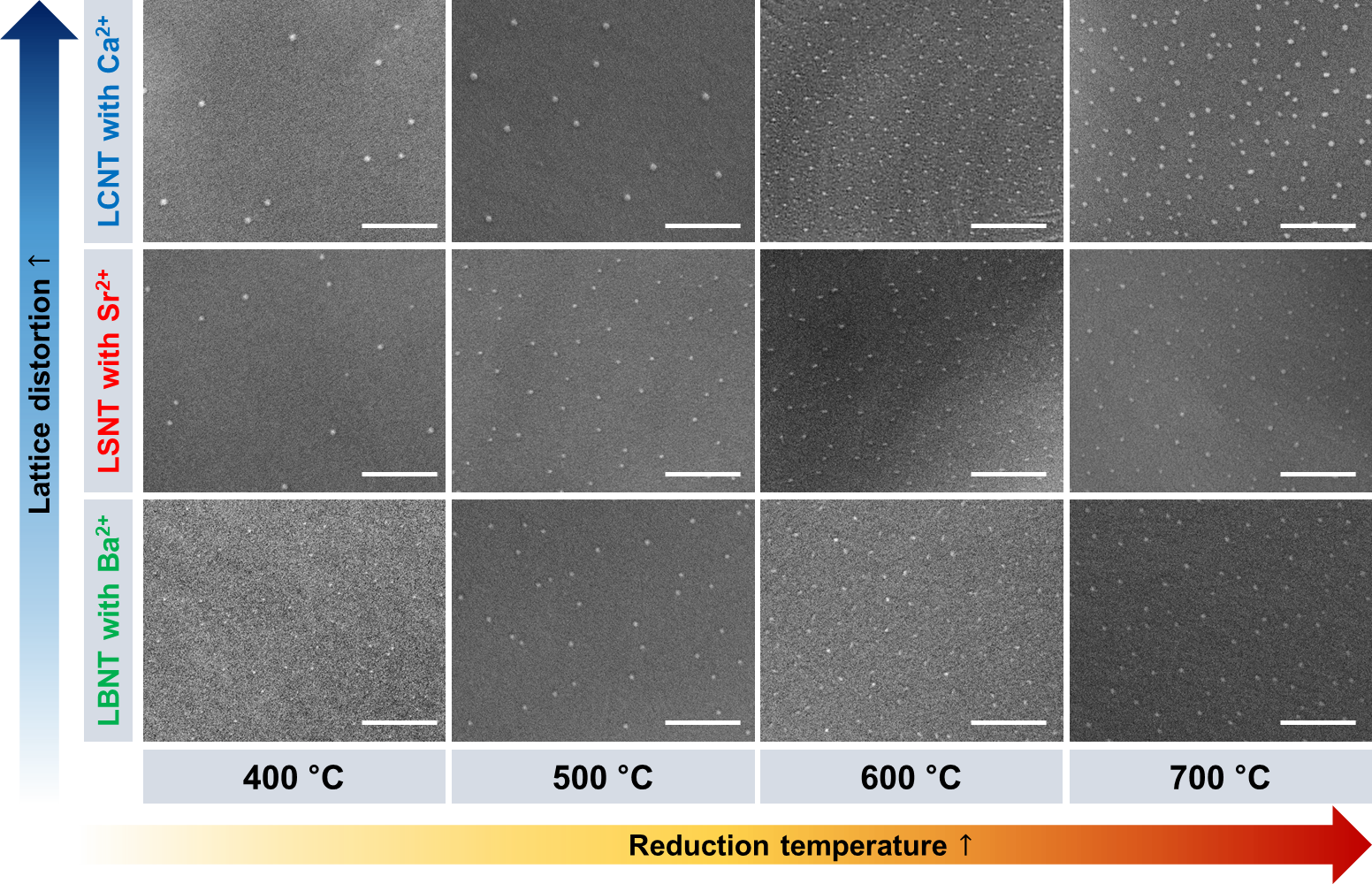


**Figure S8.** Exsolution on the perovskite oxides. Surface morphologies of LCNT, LSNT, and LBNT after reduction in pure H_2_ at 400, 500, 600, and 700 °C for 20 h. White scale bar is 200 nm.

**
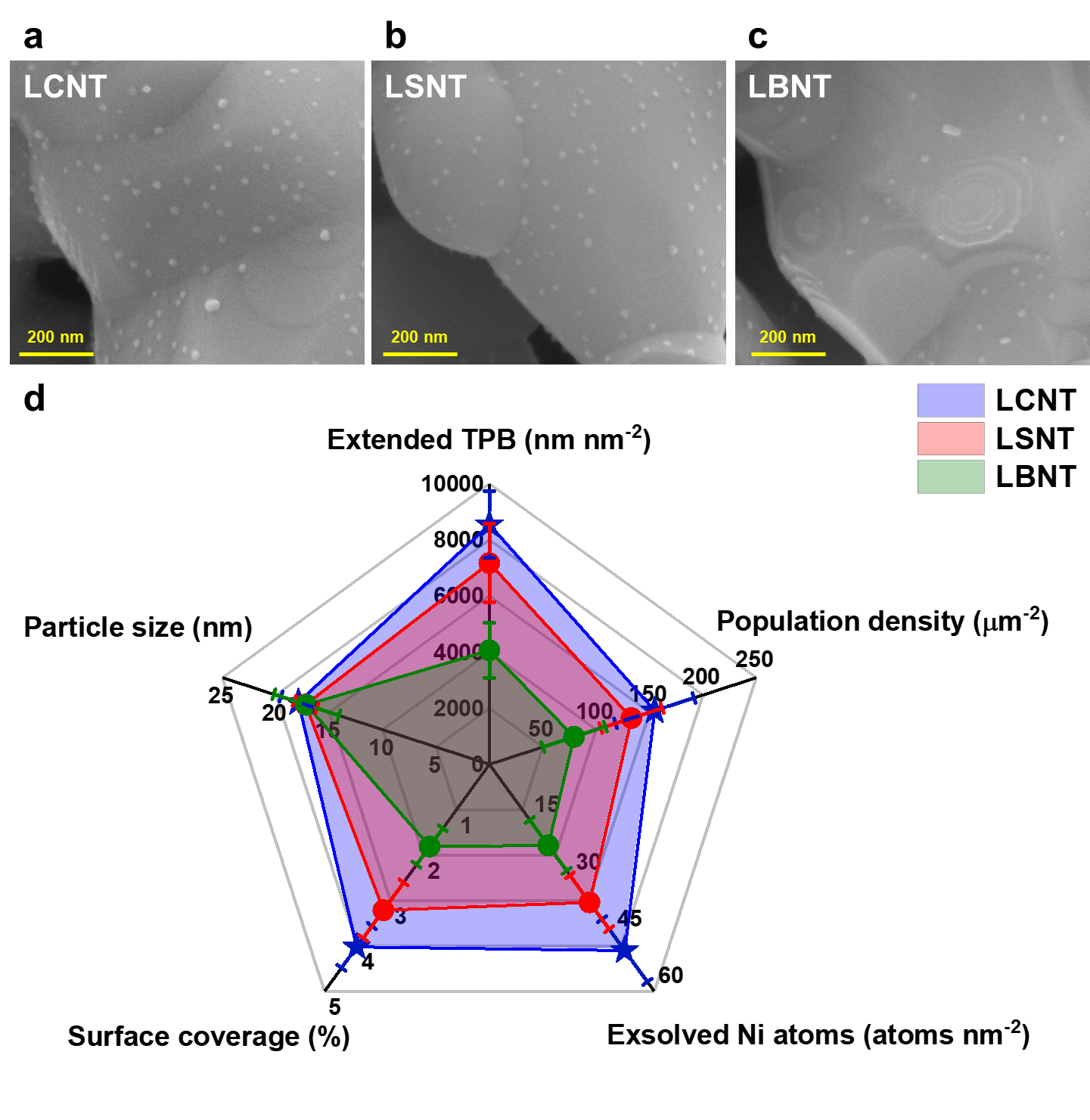
**

**Figure S9.** SEM images of (a) LCNT, (b) LSNT, and (c) LBNT electrodes reduced in H_2_ at 800 °C for 20 h. (d) Various parameters for particle analysis on the electrode surfaces, with errors expressed as standard deviations.

**
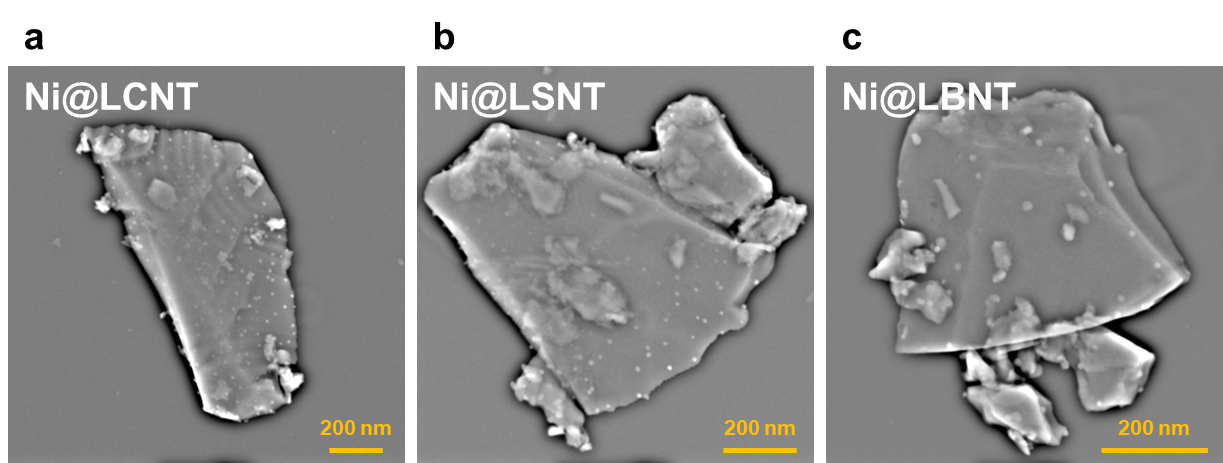
**

**Figure S10.** TEM images of (a) LCNT, (b) LSNT, and (c) LBNT catalysts reduced at 800 °C for 20 h, where Ni nanoparticles were exsolved on the perovskite oxides.


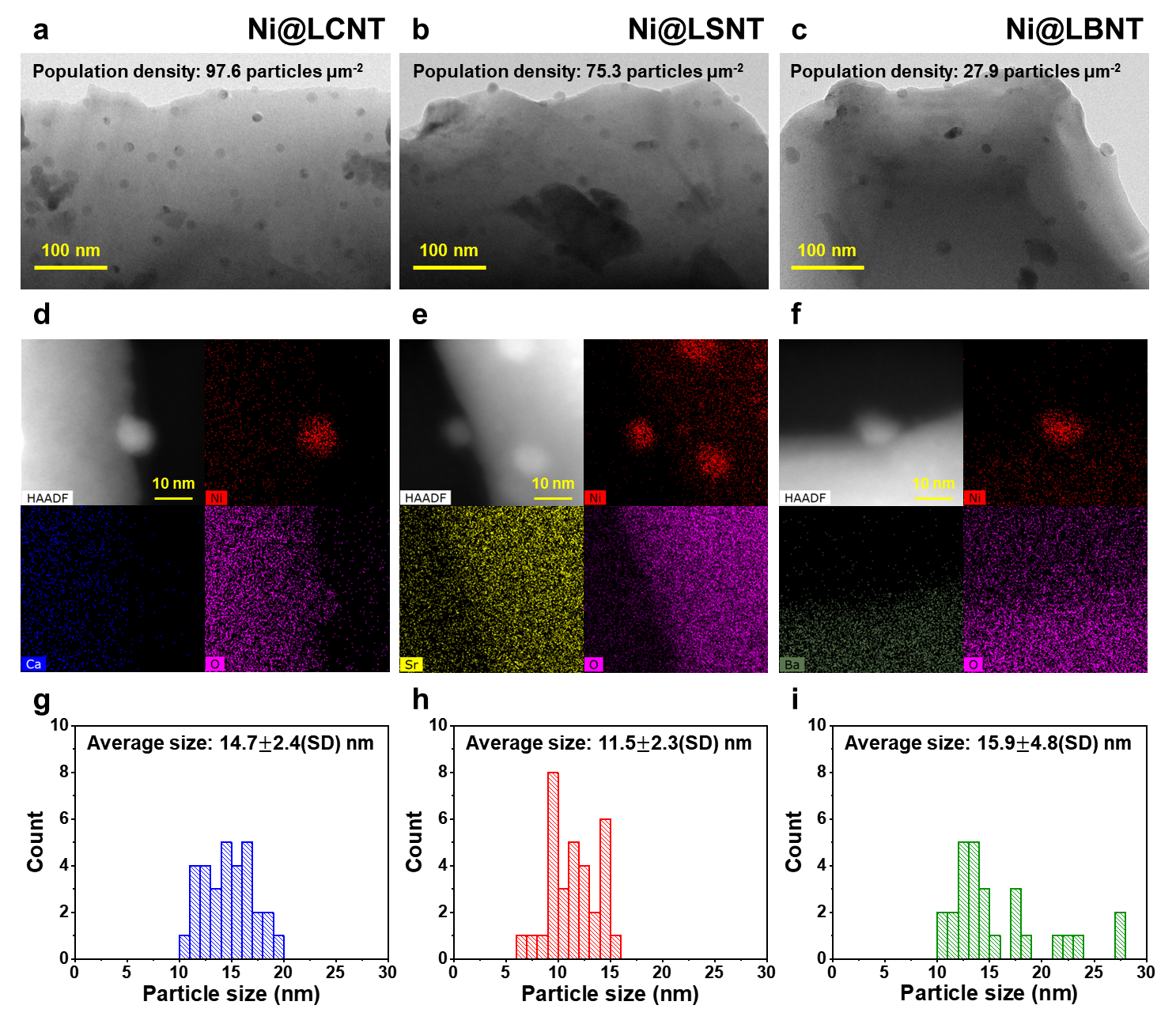


**Figure S11.** Preparation of exsolution catalysts. (a-c) FE-TEM images and (d-f) STEM/EDS analysis of LCNT, LSNT, and LBNT powders reduced in pure H_2_ at 800 °C for 20 h. (g-i) Size distribution of exsolved Ni nanoparticles on the reduced LCNT, LSNT, and LBNT (in pure H_2_ at 800 °C).

**
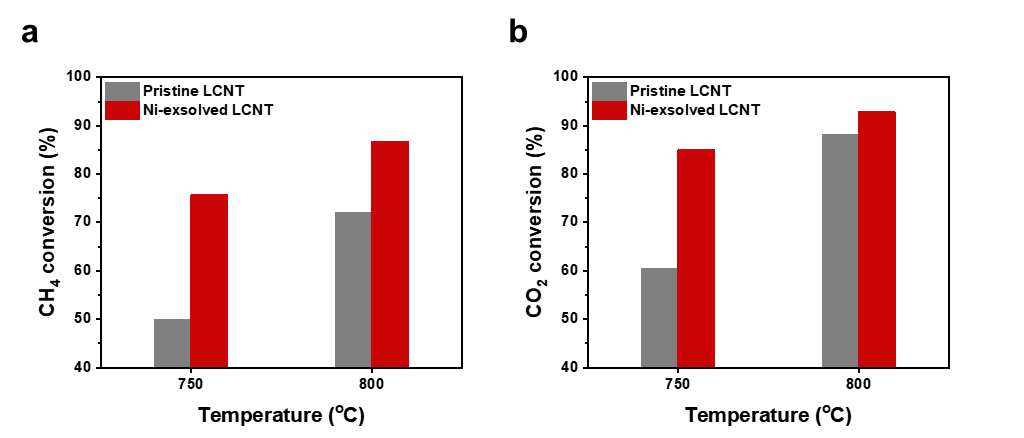
**

**Figure S12.** (a) CH_4_ conversions and (b) CO_2_ conversions of LCNT catalysts before and after reduction at 800 °C in H_2_ for 20 h.

**
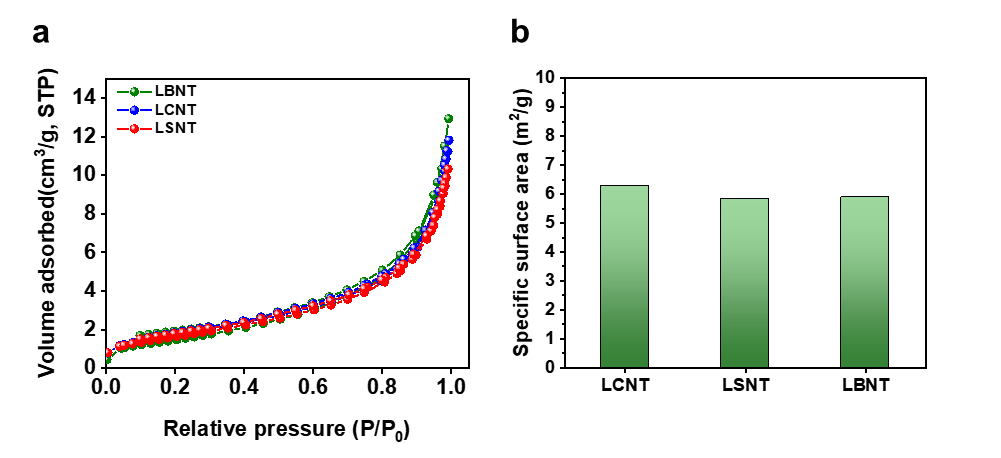
**

**Figure S13.** BET analysis of perovskite oxide catalysts. (a) N_2_ adsorption isotherm and (b) specific surface area of as-prepared LCNT, LSNT, and LBNT catalysts before reduction.

**
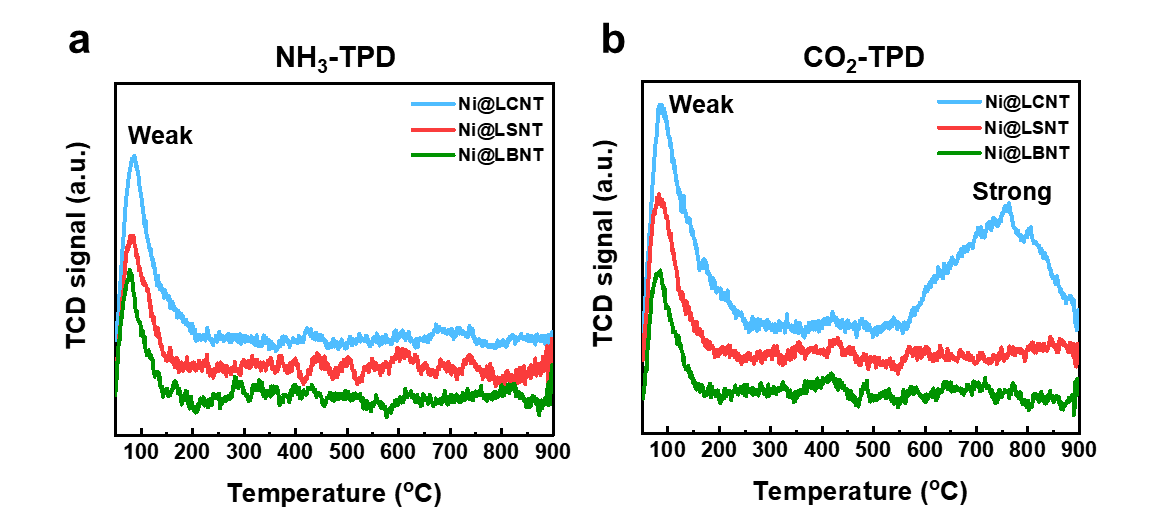
**

**Figure S14.** Surface properties of prepared exsolution catalysts. (a) NH_3_-TPD profile, and (b) CO_2_-TPD profile of 800 °C-reduced perovskite oxide catalysts.

**Table S1:** Refined crystal structure and phase information of pristine and reduced perovskite oxides.

|  | | Lattice information | | | | | | | | | | | | | | |  |
| --- | --- | --- | --- | --- | --- | --- | --- | --- | --- | --- | --- | --- | --- | --- | --- | --- | --- |
| M | Condition | | Phase | Space group | a (Å) | b (Å) | c (Å) | a' (°) | a_p_ (Å) | V_p_ (Å^3^) | Phase fraction (mol%) | χ^2^ | Mean A–O length (Å) | Mean B–O length (Å) | Tolerance factor, τ |  | |
| **Ca** | Pristine | | Perovskite | Pbnm | 5.5076 | 5.5086 | 7.7925 | 90 | 3.8953 | 236.42 | 100 | 1.217 | 2.7164 | 1.9732 | 0.973 |  |  |
|  | Reduction  @600 ℃ | | Perovskite | Pbnm | 5.5034 | 5.5235 | 7.7858 | 90 | 3.8967 | 236.67 | 98.78 | 1.986 | 2.7171 | 1.9771 | 0.972 |  |  |
|  |  |  | Ni | Fm-3m | 3.5172 | 3.5172 | 3.5172 | 90 | 3.5172 | 43.51 | 1.22 |  |  |  |  |  |  |
|  | Reduction  @750 ℃ | | Perovskite | Pbnm | 5.5202 | 5.5012 | 7.7800 | 90 | 3.8944 | 236.27 | 96.06 | 1.444 | 2.7417 | 1.9717 | 0.983 |  |  |
|  |  |  | Ni | Fm-3m | 3.5172 | 3.5172 | 3.5172 | 90 | 3.5172 | 43.51 | 3.94 |  |  |  |  |  |  |
|  | Reduction  @900 ℃ | | Perovskite | Pbnm | 5.4969 | 5.5288 | 7.77599 | 90 | 3.8948 | 236.32 | 92.05 | 2.537 | 2.7619 | 1.9649 | 0.994 |  |  |
|  |  |  | Ni | Fm-3m | 3.5172 | 3.5172 | 3.5172 | 90 | 3.5172 | 43.51 | 7.95 |  |  |  |  |  |  |
| **Sr** | Pristine | | Perovskite | I4/mcm | 5.5278 | 5.5278 | 7.8171 | 90 | 3.9087 | 238.86 | 100 | 1.276 | 2.7686 | 1.9676 | 0.995 |  |  |
|  | Reduction  @600 ℃ | | Perovskite | I4/mcm | 5.5231 | 5.5231 | 7.8253 | 90 | 3.9078 | 238.71 | 98.77 | 2.767 | 2.7693 | 1.9707 | 0.994 |  |  |
|  |  |  | Ni | Fm-3m | 3.5172 | 3.5172 | 3.5172 | 90 | 3.5172 | 43.51 | 1.23 |  |  |  |  |  |  |
|  | Reduction  @750 ℃ | | Perovskite | I4/mcm | 5.5242 | 5.5242 | 7.8336 | 90 | 3.9097 | 239.06 | 96.78 | 1.728 | 2.7674 | 1.9627 | 0.997 |  |  |
|  |  |  | Ni | Fm-3m | 3.5172 | 3.5172 | 3.5172 | 90 | 3.5172 | 43.51 | 3.22 |  |  |  |  |  |  |
|  | Reduction  @900 ℃ | | Perovskite | I4/mcm | 5.5228 | 5.5228 | 7.8312 | 90 | 3.9087 | 238.86 | 94.38 | 2.750 | 2.7654 | 1.9587 | 0.998 |  |  |
|  |  |  | Ni | Fm-3m | 3.5172 | 3.5172 | 3.5172 | 90 | 3.5172 | 43.51 | 5.62 |  |  |  |  |  |  |
| **Ba** | Pristine | | Perovskite | I4/mcm | 5.5510 | 5.5510 | 7.8572 | 90 | 3.9263 | 242.11 | 100 | 1.311 | 2.7789 | 1.9703 | 0.997 |  |  |
|  | Reduction  @600 ℃ | | Perovskite | I4/mcm | 5.5576 | 5.5576 | 7.8538 | 90 | 3.9288 | 242.58 | 98.88 | 2.457 | 2.7806 | 1.9714 | 0.997 |  |  |
|  |  |  | Ni | Fm-3m | 3.5172 | 3.5172 | 3.5172 | 90 | 3.5172 | 43.51 | 1.12 |  |  |  |  |  |  |
|  | Reduction  @750 ℃ | | Perovskite | I4/mcm | 5.5514 | 5.5514 | 7.8630 | 90 | 3.9274 | 242.33 | 97.38 | 1.760 | 2.7793 | 1.9698 | 0.998 |  |  |
|  |  |  | Ni | Fm-3m | 3.5172 | 3.5172 | 3.5172 | 90 | 3.5172 | 43.51 | 2.62 |  |  |  |  |  |  |
|  | Reduction  @900 ℃ | | Perovskite | I4/mcm | 5.5627 | 5.5627 | 7.8684 | 90 | 3.9337 | 243.48 | 95.80 | 2.597 | 2.7818 | 1.9675 | 0.9997 |  |  |
|  |  |  | Ni | Fm-3m | 3.5172 | 3.5172 | 3.5172 | 90 | 3.5172 | 43.51 | 4.20 |  |  |  |  |  |  |

**Table S2:** Reported Ni-based catalysts for DRM in the literature.

| **Catalyst** | **Preparation method** | **Temp.**  **(°C)** | **Stream**  **time (h)** | **GHSV or WHSV** | **Conversion (%)** | **Degradation rate (%/h)** | **H_2_/CO** | **Ref.** |
| --- | --- | --- | --- | --- | --- | --- | --- | --- |
| **Ni@LCNT** | **Exsolution** | **800** | **100** | **15,000 ml_CH4_ g^-1^ h^-1^**  **^­­^CH_4_:CO_2_=1:1** | **CH_4_: 84→81**  **CO_2_: 91→90** | **CH_4_: 0.036**  **CO_2_: 0.013** | **0.96** | **In this work** |
| **Ni@LSNT** | **Exsolution** | **800** | **100** | **15,000 ml_CH4_ g^-1^ h^-1^**  **^­­^CH_4_:CO_2_=1:1** | **CH_4_: 82→74**  **CO_2_: 90→84** | **CH_4_: 0.099**  **CO_2_: 0.067** | **0.94** | **In this work** |
| **Ni@LBNT** | **Exsolution** | **800** | **100** | **15,000 ml_CH4_ g^-1^ h^-1^**  **^­­^CH_4_:CO_2_=1:1** | **CH_4_: 71→66**  **CO_2_: 85→81** | **CH_4_: 0.070**  **CO_2_: 0.048** | **0.88** | **In this work** |
| **Ni@La_0.9_Mn_0.8_Ni_0.2_O_3_** | **Exsolution** | **700** | **24** | **4,000 ml_CH4_ g^-1^ h^-1^**  **^­­^CH_4_:CO_2_:He=1:1:1** | **CH_4_: 83→81**  **CO_2_: 84→81** | **CH_4_: 0.100**  **CO_2_: 0.149** | **-** | **[1]** |
| **Ni@Ba_8_Ta_6_Ni**  **O_24_** | **Exsolution** | **800** | **4** | **3,000 ml_CH4_ g^-1^ h^-1^**  **CH_4_:CO_2_:He:Ar=1:1:2:16** | **CH_4_: 40→30**  **CO_2_: 30→19** | **CH_4_: 6.250**  **CO_2_: 9.166** | **-** | [2] |
| **Ni@SiO_2_-S1** | **Encapsulation** | **700** | **28** | **18,750 ml_CH4_ g^-1^ h^-1^**  **^­­^CH_4_:CO_2_:Ar=1:1:2** | **CH_4_: 75→73**  **CO_2_: 85→80** | **CH_4_: 0.110**  **CO_2_: 0.197** | **0.84** | **[3]** |
| **Ni@SiO_2_-F** | **Impregnation** | **700** | **6** | **12,000 ml_CH4_ g^-1^ h^-1^**  **^­­^CH_4_:CO_2_:Ar=1:1:2** | **CH_4_: 62→58**  **CO_2_: 78→76** | **CH_4_: 1.075**  **CO_2_: 0.427** | **-** | **[4]** |
| **Ni@Hol S1 zeolite** | **Impregnation** | **800** | **6** | **36,000 ml_CH4_ g^-1^ h^-1^**  **^­­^CH_4_:CO_2_=1:1** | **CH_4_: 80→65**  **CO_2_: 82→75** | **CH_4_: 3.125**  **CO_2_: 1.423** | **-** | **[5]** |
| **Ni@SiO_2_** | **Wet-coating** | **750** | **25** | **12,000 ml_CH4_ g^-1^ h^-1^**  **^­­^CH_4_:CO_2_:N_2_=1:1:2** | **CH_4_: 58→54**  **CO_2_: 71→66** | **CH_4_: 0.276**  **CO_2_: 0.282** | **0.77** | **[6]** |
| **Ni@MFI zeolite** | **Impregnation** | **600** | **60** | **6,200 ml_CH4_ g^-1^ h^-1^**  **^­­^CH_4_:CO_2_:N_2_=1:1:8** | **CH_4_: 62→60**  **CO_2_: 71→65** | **CH_4_: 0.067**  **CO_2_: 0.137** | **-** | **[7]** |
| **Ni@SiO_2_** | **Impregnation** | **800** | **9** | **36,000 ml_CH4_ g^-1^ h^-1^**  **^­­^CH_4_:CO_2_=1:1** | **CH_4_: 85→78**  **CO_2_: 91→84** | **CH_4_: 0.915**  **CO_2_: 0.855** | **0.95** | **[8]** |
| **Ni@La_2_O_3_-CeO_2_** | **Wetness impregnation** | **650** | **24** | **9,000 ml_CH4_ g^-1^ h^-1^**  **CH_4_:CO_2_:N_2_=**  **15:15:70** | **CH_4_: 54→48**  **CO_2_: 61→59** | **CH_4_: 0.463**  **CO_2_: 0.137** | **0.64** | **[9]** |
| **Ni@CeO_2_** | **Impregnation** | **700** | **10** | **4,800 ml_CH4_ g^-1^ h^-1^**  **CH_4_:CO_2_:N_2_=**  **1:1:10** | **CH_4_: 81→59**  **CO_2_: 85→70** | **CH_4_: 2.716**  **CO_2_: 1.765** | **0.90** | **[10]** |
| **Ni@MgO-CeO_2_** | **Co-impregnation** | **700** | **10** | **4,800 ml_CH4_ g^-1^ h^-1^**  **CH_4_:CO_2_:N_2_=**  **1:1:10** | **CH_4_: 86→77**  **CO_2_: 89→86** | **CH_4_: 1.047**  **CO_2_: 0.349** | **1.04** | **[10]** |
| **Ni@Al_2_O_3_** | **Impregnation** | **800** | **50** | **21,600 ml_CH4_ g^-1^ h^-1^**  **CH_4_:CO_2_:N_2_=**  **9:9:2** | **CH_4_: 69→65**  **CO_2_: 75→72** | **CH_4_: 0.116**  **CO_2_: 0.080** | **0.91** | **[11]** |
| **Ni@La_2_O_2_CO_3_-Al_2_O_3_** | **Wetness**  **co-impregnation** | **650** | **50** | **36,000 ml_CH4_ g^-1^ h^-1^**  **CH_4_:CO_2_:N_2_=**  **15:15:70** | **CH_4_: 67→63**  **CO_2_: 70→68** | **CH_4_: 0.119**  **CO_2_: 0.057** | **0.86** | **[12]** |
| **Ni@ZrO_2_** | **Co-precipitation** | **750** | **60** | **12,000 ml_CH4_ g^-1^ h^-1^**  **^­­^CH_4_:CO_2_=1:1** | **CH_4_: 80→63**  **CO_2_: 85→72** | **CH_4_: 0.354**  **CO_2_: 0.255** | **0.94** | **[13]** |
| **Ni@ZrO_2_ promoted by La** | **Impregnation** | **700** | **5** | **12,000 ml_CH4_ g^-1^ h^-1^**  **^­­^CH_4_:CO_2_=1:1** | **CH_4_: 70→58**  **CO_2_: 73→70** | **CH_4_: 3.429**  **CO_2_: 0.822** | **0.95** | **[14]** |
| **Ni@Mg-Al LDH** | **Freeze drying** | **800** | **40** | **240,000 ml_CH4_ g^-1^ h^-1^**  **CH_4_:CO_2_:N_2_=**  **1:1:8** | **CH_4_: 88→70** | **CH_4_: 0.490** | **-** | **[15]** |
| **Ni@TiO_2_** | **Evaporation induced self-assembly** | **700** | **10** | **2,000 ml_CH4_ g^-1^ h^-1^**  **CH_4_:CO_2_:N_2_=**  **1:1:8** | **CH_4_: 74→68**  **CO_2_: 84→80** | **CH_4_: 0.810**  **CO_2_: 0.476** | **0.95** | **[16]** |
| **Ni@ZnO** | **Co-precipitation** | **700** | **30** | **180,000 ml_CH4_ g^-1^ h^-1^**  **CH_4_:CO_2_:Ar=**  **15:15:70** | **CO_2_: 48→41** | **CO_2_: 0.486** | **1.00** | **[17]** |
| **Ni@dBeta zeolite** | **solid-state grinding** | **650** | **100** | **95,000 ml_CH4_ g^-1^ h^-1^**  **CH_4_:CO_2_:Ar=**  **9:9:82** | **CH_4_: 49→47** | **CH_4_: 0.041** | **-** | **[18]** |

**References**

[1] T. Wei, L. Jia, H. Zheng, B. Chi, J. Pu, J. Li, LaMnO3-based perovskite with in-situ exsolved Ni nanoparticles: a highly active, performance stable and coking resistant catalyst for CO2 dry reforming of CH4, Appl Catal A Gen 564 (2018) 199–207. https://doi.org/10.1016/j.apcata.2018.07.031.

[2] T. Pussacq, O. Mentré, F. Tessier, A. Löfberg, M. Huvé, J. Guererro Caballero, S. Colis, H. Kabbour, Nanometric nickel exsolution in the hexagonal perovskite Ba8Ta6NiO24: Survey of the structural, magnetic and catalytic features, J Alloys Compd 766 (2018) 987–993. https://doi.org/10.1016/j.jallcom.2018.07.016.

[3] S. Xu, T.J.A. Slater, H. Huang, Y. Zhou, Y. Jiao, C.M.A. Parlett, S. Guan, S. Chansai, S. Xu, X. Wang, C. Hardacre, X. Fan, Developing silicalite-1 encapsulated Ni nanoparticles as sintering-/coking-resistant catalysts for dry reforming of methane, Chemical Engineering Journal 446 (2022). https://doi.org/10.1016/j.cej.2022.137439.

[4] S. Wen, M. Liang, J. Zou, S. Wang, X. Zhu, L. Liu, Z.J. Wang, Synthesis of a SiO2 nanofibre confined Ni catalyst by electrospinning for the CO2 reforming of methane, J Mater Chem A Mater 3 (2015) 13299–13307. https://doi.org/10.1039/c5ta01699a.

[5] C. Dai, S. Zhang, A. Zhang, C. Song, C. Shi, X. Guo, Hollow zeolite encapsulated Ni-Pt bimetals for sintering and coking resistant dry reforming of methane, J Mater Chem A Mater 3 (2015) 16461–16468. https://doi.org/10.1039/c5ta03565a.

[6] J. Zhang, F. Li, Coke-resistant Ni at SiO2 catalyst for dry reforming of methane, Appl Catal B 176–177 (2015) 513–521. https://doi.org/10.1016/j.apcatb.2015.04.039.

[7] Q. Cheng, X. Yao, L. Ou, Z. Hu, L. Zheng, G. Li, N. Morlanes, J.L. Cerrillo, P. Castaño, X. Li, J. Gascon, Y. Han, Highly Efficient and Stable Methane Dry Reforming Enabled by a Single-Site Cationic Ni Catalyst, J Am Chem Soc 145 (2023) 25109–25119. https://doi.org/10.1021/jacs.3c04581.

[8] Y. Zhang, G. Zhang, J. Liu, T. Li, Y. Wang, Y. Zhao, G. Li, Y. Zhang, Dry reforming of methane over Ni/SiO2 catalysts: Role of support structure properties, Fuel 340 (2023) 127490. https://doi.org/10.1016/j.fuel.2023.127490.

[9] M. Grabchenko, G. Pantaleo, F. Puleo, T.S. Kharlamova, V.I. Zaikovskii, O. Vodyankina, L.F. Liotta, Design of Ni-based catalysts supported over binary La-Ce oxides: Influence of La/Ce ratio on the catalytic performances in DRM, Catal Today 382 (2021) 71–81. https://doi.org/10.1016/j.cattod.2021.07.012.

[10] Z. Ou, J. Ran, H. Qiu, X. Huang, C. Qin, Uncovering the effect of surface basicity on the carbon deposition of Ni/CeO2 catalyst modified by oxides in DRM, Fuel 335 (2023) 126994. https://doi.org/10.1016/j.fuel.2022.126994.

[11] Y. Xu, X. Du, L. Shi, T. Chen, H. Wan, P. Wang, S. Wei, B. Yao, J. Zhu, M. Song, Improved performance of Ni/Al2O3 catalyst deriving from the hydrotalcite precursor synthesized on Al2O3 support for dry reforming of methane, Int J Hydrogen Energy 46 (2021) 14301–14310. https://doi.org/10.1016/j.ijhydene.2021.01.189.

[12] K. Li, C. Pei, X. Li, S. Chen, X. Zhang, R. Liu, J. Gong, Dry reforming of methane over La2O2CO3-modified Ni/Al2O3 catalysts with moderate metal support interaction, Appl Catal B 264 (2020) 118448. https://doi.org/10.1016/j.apcatb.2019.118448.

[13] M. Zhang, X. Zhou, J. Yang, T. Yang, Z. Liu, Y. Han, Deciphering the ZrO2 phase engineering effects on dry reforming of methane over the Ni/ZrO2 catalysts, Fuel 349 (2023) 128705. https://doi.org/10.1016/j.fuel.2023.128705.

[14] M. Zhang, J. Zhang, Z. Zhou, S. Chen, T. Zhang, F. Song, Q. Zhang, N. Tsubaki, Y. Tan, Y. Han, Effects of the surface adsorbed oxygen species tuned by rare-earth metal doping on dry reforming of methane over Ni/ZrO2 catalyst, Appl Catal B 264 (2020) 118522. https://doi.org/10.1016/j.apcatb.2019.118522.

[15] J. Huang, Y. Yan, S. Saqline, W. Liu, B. Liu, High performance Ni catalysts prepared by freeze drying for efficient dry reforming of methane, Appl Catal B 275 (2020) 119109. https://doi.org/10.1016/j.apcatb.2020.119109.

[16] M. Shah, M.K. Al Mesfer, M. Danish, Effect of titania synthesis conditions on the catalytic performance of mesoporous Ni/TiO2 catalysts for carbon dioxide reforming of methane, Int J Hydrogen Energy 47 (2022) 8867–8874. https://doi.org/10.1016/j.ijhydene.2021.12.246.

[17] J. Niu, S.E. Liland, J. Yang, K.R. Rout, J. Ran, D. Chen, Effect of oxide additives on the hydrotalcite derived Ni catalysts for CO2 reforming of methane, Chemical Engineering Journal 377 (2019) 119763. https://doi.org/10.1016/j.cej.2018.08.149.

[18] J. Zhang, Y. Li, H. Song, L. Zhang, Y. Wu, Y. He, L. Ma, J. Hong, A. Tayal, N. Marinkovic, D.E. Jiang, Z. Li, Z. Wu, F. Polo-Garzon, Tuning metal-support interactions in nickel–zeolite catalysts leads to enhanced stability during dry reforming of methane, Nature Communications 15 (2024). https://doi.org/10.1038/s41467-024-50729-8.
